# Improving smallholder agriculture via video-based group extension^☆^

**DOI:** 10.1016/j.jdeveco.2024.103267

**Published:** 2024-06

**Authors:** Tushi Baul, Dean Karlan, Kentaro Toyama, Kathryn Vasilaky

**Affiliations:** a Playgig, United States of America; b Northwestern University, Innovations for Poverty Action, and M.I.T. Jameel Poverty Action Lab, United States of America; c University of Michigan, School of Information, United States of America; d Cal Poly, Department of Economics and Columbia University, Intl Res Inst Climate & Society, United States of America

**Keywords:** Water, Field experiment, Agriculture, System rice intensification, Video-based training, Group extension, information

## Abstract

Providing agricultural advice at scale poses operational challenges. Technology may help if repeating content reinforces learning for recipients and thus improves adoption, but risks reducing efficacy given limited customization and human interaction. We tested videos shared with female farmers in India as a supplement to standard human-provided extension services promoting a climate-smart practice, System Rice Intensification. The average treatment effects are large but imprecise because of non-normally distributed outcomes, specifically fat right tails. Weighted quantile regressions show that the imprecision in estimating an average treatment effect comes from farmers with output or yields in the upper quantiles. Both quantile regressions of the 25% and 50% quantiles and a Bayesian hierarchical model (robust to several priors) reveal positive treatment effects, and two subtreatments, one that reinforces information on labor costs from adoption and a second that presents role models to motivate adoption, lead to even higher estimated treatment effects on output.

## Highlights

• Poverty implies.
• freedom to decide, to dream.
• To decide to dream.
• For highlights beyond this 5-7-5 haiku, please read on.

## 1 Introduction

To be effective, information interventions such as agricultural extension programs must not only deliver relevant content but must also deliver that content well. Evaluations of extension efforts yield a wide range of treatment effects, yet most evaluations focus on varying the content (typically testing content versus no content) and not pedagogical aspects (such as complementing human interaction with videos versus just human interaction). Furthermore, the decision to shift to technology, either in lieu of or to complement human interaction, may be critical for testing how and what information is disseminated. Video, in particular, offers the opportunity to control the message, by both repeating (i.e., reinforcing) content to recipients, ensuring consistency across recipients, and providing a level of relatability if presented by protagonists in similar circumstances to their recipients. Yet technology also risks favoring control and homogeneity of messaging in place of the customization inherent from human interaction.

We test technologically-distributed agricultural extension in India by supplementing an existing face-to-face program with a video based component in a randomized controlled trial (RCT). The videos include multi-stepped instructions on a climate-smart technology, system rice intensification (SRI), and aim to increase adoption by reinforcing the face-to-face extension with both some repetition of material combined with more nuanced messages addressing common perceived challenges particularly for female farmers. We conducted the RCT with Digital Green (DG), a non-governmental organization recognized for its tailored video instruction aimed at hard-to-reach female farmers in rural Bihar, India. The experiment compares traditional face-to-face training without videos to traditional training complemented by videos, and does not include a pure control group. The core content remains consistent across all participants, as National Rural Livelihoods Mission (NRLM) extension training was already active in all rice-growing districts of Bihar.^1^ The addition of video aims to enhance extension training by ensuring quality control, fostering message consistency, and tailoring information to the specific uncertainties related to the SRI practice — labor supply and personal self-efficacy. To estimate the effects of layering video content onto traditional extension training, we compare our control group, farmers who receive NRLM training, to our treatment groups — farmers who receive NRLM and video training. To estimate the effects of targeted messaging regarding labor supply and personal self-efficacy, we compare our control group to farmers who receive DG videos that also include messages on labor supply, self-efficacy, and both labor supply and self-efficacy. The labor message prepares farmers for the reality of the labor requirements, while the self-efficacy message helps participants feel capable of implementing the technique.

The average treatment effects are economically large and positive for output and yields along the main DG arm (DG no messaging) but are not statistically significant after applying multiple hypothesis corrections. However, a challenge with our outcome variables, including output, yields, inputs (labor and expenditures), and estimated profits, is that their distributions exhibit fat tails. Fat tails may be due to measurement error, population characteristics, or heterogeneous treatment effects, and are common in agricultural data (Okorie et al., 2023). Heavy-tailed data can complicate estimation of average treatment effects because the standard assumptions for average treatment effect do not apply. Quantile regressions are often the next step at providing a more complete picture of treatment effects (Meager, 2022, Li, 2015, Li et al., 2023), but estimating treatment effects in the upper quantiles is complicated due to data sparseness in the tail area (Li et al., 2023), and estimating quantile effects in the lower quantiles is complicated by large spikes in the data around zero (Meager, 2022). We thus take a broader methodological perspective in estimating the impacts of our intervention that includes standard ATEs, quantile regressions, weighted quantile regressions (Athey et al., 2023) and a Bayesian hierarchical model with varied priors.

First, quantile regressions demonstrate that there are economically meaningful and statistically significant effects for the 25%, and 50% quantiles on output and yields. Depending on the specification, both the main DG arm without messaging and DG arm with both messages improve output per farm and per acre. The estimates from the upper quantiles are most imprecisely measured, and this leads to imprecise estimates of the average treatment effect. To see this, we apply a weighted average quantile effect estimator (WAQ) (Athey et al., 2023), which allows for aggregation of treatment effects where the weights differ across quantiles. The WAQ estimator confirms that the observations above the median are particularly noisy and underweights the effects in the upper tails for a more precise average treatment effect. Finally, we estimate a Bayesian hierarchical model with two different sets of priors, which allows us to account for fat tails by specifying the data generating distribution. The magnitude of the mean of the Bayesian posteriors of the treatment effects on output and yield are largely in line with the magnitude of the point estimates between the 25% and median quantile regressions and both suggest that the effects on output per farm and per acre are non-zero. The median quantile effect for output per farm increases by 15% and per acre by 18%. The latter estimations indicate that our more nuanced messages regarding labor and self-efficacy have a complementary effect. The two messages together produce the most favorable results for participants’ output at the 25% quantile and median effects. For yields, the DG no messaging arm and DG + both arm are equally effective for the 25% and median quantile. In contrast, the DG arms with just one of the messages – labor or self-efficacy – did not have statistically significant impacts, and for the self-efficacy message the effect sizes were close to zero, suggesting that delivering a self-efficacy message alone actually lessened the effectiveness of the DG video. This may be because improving self-efficacy can also reduce preparedness, which is necessary for a multi-step task like SRI. Overall, our results suggest that the imprecisely measured ATE is likely due to imprecisely measured treatment effects in the upper tails as well as to heterogeneity in the treatment effects, but that there are clear economically and statistically significant effects for farmers with output at or below the median.

Our findings contribute to several literatures. First, there is an emerging literature on video-based learning in a number of non-agricultural settings that have had positive outcomes, for example: affecting life-changing behaviors such as intake of fortified salt (Banerjee et al., 2017); sexual behavior (Banerjee et al., 2019); entrepreneurial activities (Bjorvatn et al., 2020); financial literacy (Coville et al., 2019, Berg and Zia, 2017); political participation (Mvukiyehe, 2018); and conflict resolution (Levy and Green, 2009).^2^ Video has the advantage of being low cost, while still being able to target its content to the skill level of the student (Muralidharan et al., 2019) and the teacher (Jackson and Makarin, 2018). Given the success of video-based learning in other domains, it is not surprising that there has been a push for video-based training in agriculture (Fabregas et al., 2019).

We test the video medium in agriculture and its ability to relay information about the labor required for the SRI practice, and uncertainties regarding farmers’ beliefs in their own abilities. Conveying and transferring details requires an immersive experience and Digital Green accomplishes this in several ways. Their video viewing is embedded within a practice of self-help group meetings, in the same way that Jackson and Makarin (2018) and Muralidharan et al. (2019) embed video-based interventions within teacher-run instructional contexts. Information is disseminated in a “bottom-up” way and in a context that encourages discussion, similar to farmer field schools, which are typically seen as more effective than the “top-down” approach of Training and Visit (T&V) programs, but often prohibitively expensive (Anderson and Feder, 2007). A Village Resource Person (VRP) plays the videos (three in total across the season) curated by Digital Green within a practice of self-help group meetings, with a question and answer period following each video showing. The how-to videos also contain an element of entertainment – in the vein of Riley (2022), Bjorvatn et al. (2020), Banerjee et al. (2017), and Banerjee et al. (2019)– with music and a narrative arc that contain characters and a plot.

Only a handful of studies have tested the use of videos in agriculture extension training, and they all focus on adoption of practices as opposed to productive farm outcomes, as we do here. Nakasone and Torero (2016) test whether exposing children to agricultural how-to videos at school affects their parents’ knowledge of agricultural practices and outcomes. They find that parents’ knowledge increases by 26%–34% and adoption of the tested practices increases by 14%–18%. LaRochelle et al. (2017) use text messages and a video-enabled phone technology to deliver agricultural extension information. They find that a system of three weekly text messages over the growing season increases knowledge of integrated pest management (IPM) practices by 18.2 percentage points and adoption of IPM by 6.8 percentage points. Hörner et al. (2021) shows that videos added on to a “farmer-to-farmer” extension program facilitates adoption of practices related to Integrated Soil Fertility Management (ISFM), particularly for farmers who are not members of farmer groups.

Second, the delivery of the DG videos contain qualitative aspects that makes the implementation of SRI potentially less risky. In particular, video can help mimic a learn-by-doing and learn-from-others approach. Learning from peers has been to shown to be one way in which farmers improve their practices and productivity (Conley and Udry, 2010, Kondylis et al., 2017), so much so that there are models of extension training based precisely on peer-to-peer learning. Beaman et al. (2021) shows that using centrally located farmers, in terms of local social networks, is an effective means of disseminating information and increasing adoption. Hörner et al. (2021) shows that farmer-to-farmer extension training, in which extension agents train model farmers and model farmers train farmer group members, increases the number of practices adopted for Integrated Soil Fertility Management (ISFM), and increases complete adoption of all practices by 8.4 percentage points. However, there is a downside of relying on peer learning for information diffusion. Peer-to-peer learning requires a full growing season for farmers to observe their peers’ processes and outcomes from applying a technique. Not only is that a long time to wait, but those observations can be confounded by poor weather, poor soil conditions and other environmental factors. Conversely, videos can expedite the learning process and resolve some of the uncertainty in implementing a new technique when delivered by a more neutral, yet relatable, peer.

Third, we add to the growing literature in economics addressing fat-tailed data (Lewis and Rao, 2015, Crépon et al., 2019, Azevedo et al., 2020, Stoyanov et al., 2011), and studies that employ multiple methods to address this issue (Meager, 2022, Meager, 2019, Dehejia, 2003, Vivalt, 2020). Fat tails can lead to underpowered studies even with very large sample sizes (Azevedo et al., 2020, Lewis and Rao, 2015). Our work demonstrates that other studies investigating noisy outcomes such as output and revenue should review the distribution of their data, and consider that not accounting for fat tails could result in erroneous conclusions.

Three additional studies, by Abate et al. (2023), Lecoutere et al. (2023) and Van Campenhout et al. (2020), also investigate the impact of DG’s methodology (in Ethiopia and Uganda). Abate et al. (2023) focuses on adoption of various practices when husbands are present at training across several crops as the main outcome, where we focus on production and productivity. Overall, they find that DG improves adoption beyond traditional T&V visits (ranging in increases of 3 to 10 percentage points above the control group – a similar magnitude found in this study). Spousal presence, however, did not affect adoption rates. Van Campenhout et al. (2020) examines impacts on yield compared to a pure control with no training and finds productivity increases of 10%, but their added treatments of interactive voice messages and short messages services (SMS) that remind farmers about agronomic practices had no added effect. Lecoutere et al. (2023) focuses on knowledge acquisition and who receives information in the household, as well as on production and productivity. There is a statistically significant increase in women’s knowledge index when women receive information alone or in pairs, while men’s knowledge index exhibits a statistically significant decrease when women are present. Women’s production and productivity also exhibit a statistically significant increase on their own plots (and no effect on jointly managed plots) when trained alone, 35.8 kg (90% increase over the control) and 50.4 kg/acre respectively (an 88% increase over the control), or in pairs, 51.5 kg (130% increase over the control) and 75.1 kg/acre (131% increase over the control) respectively .

## 2 The setting: Bihar, Digital Green and system of rice intensification

We conducted the study in three districts in Bihar, India: Nalanda, Muzzaffapur, and Purnia. Bihar is one of the poorest states in India, with a per capita GDP of 28,317 Rps (440 USD) per year, or about 1.20 USD per day, and low literacy rates: 69% for males and 49% for females.^3^ This is particularly relevant as many of the latest extension trials use short message services (SMS), which requires that farmers own their own phones and are literate. This frequently excludes female farmers from being able to access agricultural extension information (Kansiime et al., 2019). In terms of socioeconomic status, the women are all from reserved classes — namely, those classified as Scheduled Castes (SCs), Scheduled Tribes (STs), and Other Backward Classes (OBCs). This system of reserved classes is meant to address historical injustices and inequalities perpetuated by the caste system. For example, the Yadav, a backwards class, is comprised of peasant-pastoral communities that are primarily landless, agricultural laborers. They are one of the most discriminated caste group in the feudal society of rural Bihar. Social norms can prevent any training from reaching these classes, particularly women (Krishna et al., 2019). In addition, women are generally placed below men and are denied an equal footing with men in society across many areas of life including access to food and nutrition, education, health and economic opportunity (Pankaj, 2020).

Agriculture remains the primary livelihood of individuals in Bihar, and is key to improving individual well being and general food security. Yet, even during the Green Revolution, Bihar experienced some of the lowest poverty reduction (Ravallion and Datt, 2002). With increasing weather extremes, particularly in Bihar, rainfed agricultural region is subject to more agricultural shocks (World Bank, 2023, Samantaray and Gouda, 2023, United Nations Convention to Combat Desertification, 2022). Water productivity has been steadily declining for rice production in Bihar (Najmuddin et al., 2018). Agricultural extension training in India is meant to support farmers in this ever-changing climate, but extension training is highly dispersed, which can make it difficult to quantify who and how many people are being reached. Among the relevant bodies are the Indian Council for Agricultural Research (ICAR), which provides training information to the Department of Agriculture (DoA); the state agricultural universities (SAU), which works with ICAR on relevant research topics; the Agriculture Technology Information Centres (ATICs) and Krishi Vigyan Kendras (KVKs, or Farm Science Centers), where ICAR conducts its research; and the Agricultural Technology Management Agency (ATMA), a central government initiative that supports decentralized state extension programs.^4^ Most of these institutions follow a T&V structure, which are top-down, face-to-face, and insufficiently resourced to meet demand. As of 2017, the ratio of public extension workers to families has been 1162:1.^5^

The above context presents itself as one in which video based training on SRI directed at female farmers can be impactful. First, the videos are created with female protagonists of the same caste as their viewers, which has been shown to improve uptake of technologies (Raghunathan et al., 2023), and SRI requires less water and is less sensitive to droughts (Sridevi and Chellamuthu, 2012, Mishra and Salokhe, 2010). The ability to include short but direct messaging on labor and self-efficacy is also crucial. But farmers may be hesitant to invest in that increased cost of labor if they are uncertain about the returns to SRI. The message reduces some of this uncertainty in terms of how much labor is needed and the potential returns. Furthermore, given women’s status at home and in the agricultural system, they are less likely to feel capable of implementing a more complex technique and the self-efficacy message addresses that.

### 2.1 Digital Green & NRLM

Microsoft India created DG in order to develop a technological solution that provides effective agricultural extension in India at scale. It began as a Microsoft research project whose early promise in India led to spinning off the effort in 2008 as a non-profit. DG focuses its efforts on amplifying the impact of smallholder extension programs. It accomplishes this by overseeing the production of short how-to videos featuring local farmers executing productive agricultural practices. The videos are then used as the basis for mediated group instruction, which involves frequent pauses during video playback with a facilitator asking questions of the audience. DG partners with and trains other organizations that disseminate the techniques, and its method is also now used for other types of behavior change campaigns, such as in health and nutrition. The video content is generated using an iterative approach with the agricultural organizations and farmers for whom they are developed. A few key aspects of DG’s early qualitative fieldwork found that viewers prefer to see and hear information coming from individuals similar to themselves as opposed to trainers or government officials; they prefer seeing multiple people and multiple locations throughout the video; and they prefer mediation with pauses and interaction (Gandhi et al., 2009). Gandhi et al. (2009) was a small-scale study that suggested that Digital Green was 10 times more cost-effective than T&V in persuading farmers to adopt a new practice. As of 2018, Digital Green videos have been viewed by over 1.5 million farmers in India, Ethiopia, and a handful of other countries.

In 2011, India’s Ministry of Rural Development launched the National Rural Livelihood Mission (NRLM), a program supported by the World Bank. In Bihar, NRLM established a semi-autonomous body called Jeevika, which was state-funded and organized as a non-profit NGO. Its aim was to establish a network of self-help groups (SHGs) in which at least one woman from every poor rural household was a member of a small group of 10–20 individuals that met regularly. Jeevika would then involve the SHGs in microcredit programs, health and nutrition education, and agricultural extension.^6^ Following a common practice among Indian NGOs, Jeevika implemented agricultural extension within the SHG structure, with extension officers providing agricultural advice and fielding questions at SHG group meetings, as opposed to the one-on-one visits that are common with T&V. DG’s methodology of group video-viewing fits well with SHG mobilization. Jeevika committed to incorporating the methodology across Bihar with staggered phase-in, so as to permit a RCT. In the control and treatment villages, Jeevika implemented in-person extension via SHGs and locally hired facilitators. In treatment villages, Jeevika, with advice from DG, ran video-viewing sessions led by facilitators.

### 2.2 System of rice intensification

Rice, or paddy, as it is sometimes known, is a water-intensive crop that is grown widely in both flood- and drought-prone Bihar. Conventional rice cultivation consists of continuous flooding of fields with groundwater. However, India is rapidly depleting its scarce groundwater resources in semi-arid areas of India, as much as a foot per year (Russo et al., 2015, Fishman et al., 2015, Rodell et al., 2009). Roy et al. (2016) show that between 1901 and 2002 rainfall has declined by 1.974 mm per year and mean temperature has increased by 0.479 degrees Celsius per year in Bihar. The Ministry of Water Resources has reported levels of arsenic (above 10 ppb) and fluoride (more than 1.5 mg/l) beyond the permissible limits in Bihar, a direct consequence of groundwater depletion.^7^ In addition, Bihar is one of the worst ranked states in India in terms of water conservation (a water index below 50%) according to the Composite Water Management Index (CWMI) developed by the government’s National Institution for Transforming India.^8^ Yet, Bihar is a major contributor to agricultural output, and, therefore, to India’s food security. This set of circumstances – increasing groundwater depletion and food insecurity – is not unique to Bihar. All across India and other parts of the world, a changing climate will stress agriculture with more extreme storms, more intense periods of agricultural drought, and more erratic weather patterns (Collins et al., 2013, Dell et al., 2012, Adams et al., 1999, Dinar et al., 1998). Without additional training, only farmers who have the means to deepen their well and purchase stronger pumps will be able to continue to cultivate lucrative crops, such as rice, that require intensive watering.

For this reason, with input from Jeevika, we chose to focus on the introduction of the System of Rice Intensification (SRI) practice, a climate-smart technique for cultivating rice that reduces resource use while also increasing yields. The increase in yields can be quite large, in the order of 20%–100%.^9^ Importantly, SRI reduces the need for many of the resources that are typically intensively used for rice cultivation, including a reduction in irrigation, seeds, and fertilizer (Wu and Uphoff, 2015).

Typically, SRI is comprised of raising seedlings in a nursery; transplantation of seedlings of 8–14 day old seedlings; widely spaced transplants of 8–10 inches, regular weeding; and controlled water management. However, as Glover (2011) emphasizes, SRI is not a fixed technology and farmers can adopt parts of the practices on part of their field. Barrett et al. (2021) shows in a RCT of SRI with 5,486 farmers in Madagascar that treated farmers adopted only 1.3–1.74 of the six SRI practices introduced, and many farmers only applied SRI practices to part of their land. But the main differentiating factors among all the above practices tend to be controlled irrigation (as opposed to continuous flooding), which can lead to additional weeding (as seedlings compete with weeds), and the process of transplantation. Transplantation allows seedlings’ roots to grow larger and deeper into the soil that is kept well aerated. The latter changes are typically reflected in farmers’ labor costs — 62% of the extra labor for SRI is needed for weeding, and 17% for transplanting (Takahashi and Barrett, 2013, Rakotomalala, 1997). These additional labor costs can often be prohibitive in the adoption of SRI. For this reason, its profitability hinges on output prices being no lower than the price of traditionally grown rice (Alem et al., 2015), and on labor costs not being prohibitively expensive (Takahashi, 2013). In our context, market prices for rice in India are relatively stable as the government of India fixes the central procurement price of rice before planting begins (Aditya et al., 2017). This reduces some of the uncertainty in projecting potential returns. Thus, we focus on the remaining factors that we identified as impeding adoption.

## 3 Data and design

We conducted an RCT to determine the causal impact of DG by randomly assigning which villages would be offered the DG viewings of a relatively new agricultural technique, System Rice Intensification (SRI).^10^ At the start of the intervention about a third of households in the baseline had heard of SRI, and 10% had reported that they had implemented some form of SRI, but we did not confirm what had actually been implemented. (These figures are based on our baseline data, which was compromised; discussion below.)

We began with a list of 607 villages in three districts in the state of Bihar acquired via a pre-censusing of the area: Nalanda, Purnia and Muzzaffapur. The villages in these districts were selected as areas where NRLM was present, but DG had not yet been introduced nor had SRI training been conducted prior to the start of the intervention. We then randomly selected a sample of 420 villages from that list. Within each village we attempted to survey approximately six women, randomly sampled from each village’s SHG. This met the requirements for sample size based on a frequentist approach. Our frequentist power calculations were based on a 0.1 standard deviation minimum detectable effect size, 90% power, 40% correlation between baseline and follow-up measurements, and 40% between repeated follow-up measurements, with clustering at the village level and an intraclass correlation coefficient of 0.05.

All villages – in treatment and control – received NRLM training that incorporated SRI messaging, where the implementation of the rural livelihoods program in Bihar is done by an entity called Jeevika. Thus, there were no differences in the agricultural information provided across control and treatment villages. The household survey collected information on SRI adoption, household demographics, plot and paddy cultivation details, land ownership, familiarity with SRI, perceptions on costs of SRI, paddy practices, water sufficiency, access to agricultural extension, expenditures, self-efficacy and aspirations. We randomly assigned two-thirds of the villages (280) to the main treatment arm (video add-on to in-person training). Of these 280, villages were randomly (70 villages each) assigned to one of four subtreatments: base video, self-efficacy message, labor-cost message, both self-efficacy and labor-cost message. The remaining 120 villages were assigned to the control (standard NRLM in-person training with no DG video add-on).

**Fig. 1:**
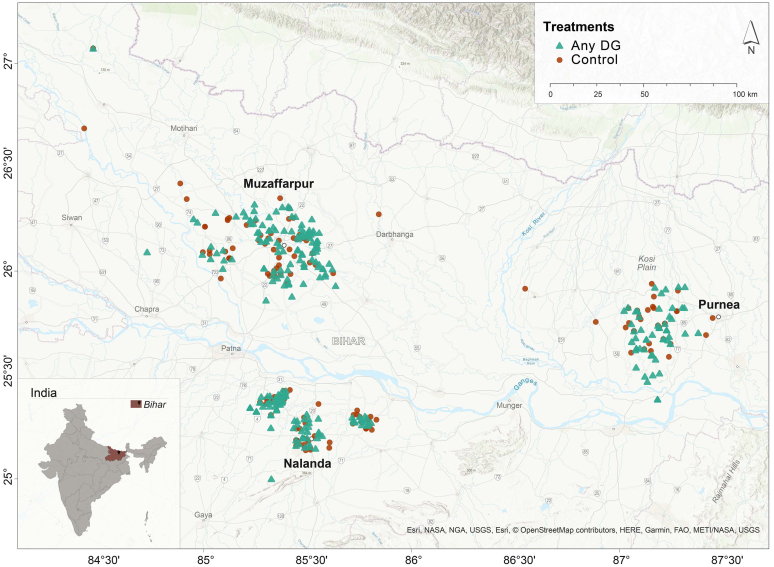
Map of treatment and control villages.

**Fig. 2:**
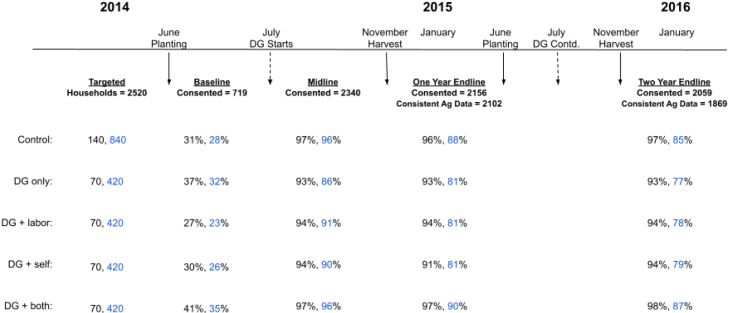
Experimental design and sample size (Villages, Households). The baseline was compromised due to mud slides and difficulty reaching nearly half of the targeted sample. Consented refers to households that we were able to contact and consented to be interviewed. Consistent Ag Data refers to the number of households whose self-reported output values cross-checked between their total reported output and their disaggregated output in terms of sold, consumed, stored, and shared paddy within a margin of error of 100 kilograms. We control for this consistency with a binary indicator in all of our regressions. We also test whether the intervention affected farmers’ consistency in reporting yields. We test whether the intervention had a statistically significant effect in explaining reporting inconsistencies (= 1 if there was a reporting inconsistency and = 0 otherwise) for yields and find no evidence of this (F-stat p-value = 0.46).

Fig. 2 depicts the experimental design and timelines. Fig. 1 depicts the placement of the control and treated villages. We collected four rounds of data (a baseline and three follow-up waves).^11^ The baseline began in May 2014 before the permissible transplantation date of June 15th in India. Note that farmers do not have to transplant on that date. It is simply the date from which they can begin to transplant, and was fixed to save water in the state. Note that NLRM/Jeevika still sets their own dates for training based on the season and their relationship with farmers. Digital Green worked in tandem with Jeevika to follow the correct timeline for training farmers. In the year the trial was conducted, seed treatment and nursery activities occurred the first and second week of July, and transplantation activities occurred in the 3rd week. The baseline survey was met with considerable attrition due to the general elections in India and floods in Bihar that year, and ultimately was incomplete, with only 719 of the 2520 households surveyed (see first row of Fig. 2). In particular, because of floods in Purnea and drought in Nalanda and Muzzaffapur, 67, 78, and 93 percent of the villages in Purnea, Nalanda and Muzzafurpur could be reached respectively, which left us with only 719 households.

We conducted the first follow-up survey in August 2014, and reached 2340 of the 2520 households surveyed. This follow-up focused on adoption practices during the 2014 kharif (summer) season, as well as inputs used during the planting season. The second follow-up survey was conducted after the season, in order to also capture agricultural outputs (spring Feb–March 2015, 2156 of the 2520 households surveyed). The third and final follow-up survey was conducted one year after the second (Feb–March 2016), in order to capture both adoption and agricultural outcomes the following year, i.e., to measure the persistence of any changes. We reached 2059 out of the 2520 households. The two end-of-season follow-up surveys included questions on inputs used during harvest, off-farm labor performed after the season ended, and perceptions regarding the SRI practice.

### 3.1 Program and intervention

The intervention took place between June and August 2014, and June and August 2015, during the months of cultivation and harvest. In the control villages, Jeevika ran its SHG-based non-video extension program. This program covered three of the main SRI topics: seed treatment, nursery bed preparation, and transplantation. Jeevika covered all three of these components of SRI verbally in one session sometime between May and June and then conducted three separate field demonstrations for each topic in the ensuing weeks. A VRP was assigned approximately 10–12 SHGs per village. For the control, VRPs used charts and posters during meetings, and utilized physical inputs (land, seeds, fertilizers) to conduct field demonstrations.

In the treatment villages, we conducted the same in-person extension training as in the control (both the verbal and demonstration plot sessions), and, in addition, Jeevika, with assistance from DG, prepared videos on the same SRI topics: seed treatment, nursery bed preparation, and transplantation.^12^ The videos were prepared based on standard DG protocol of featuring a local farmer who demonstrates the technique. Table B.1 describes the content of the videos, all involving a woman farmer between 30–45 in age. In each treatment village, Jeevika held its standard verbal session overviewing all four techniques in the treatment villages, and then VRPs held viewing sessions for each of the topics in the following weeks. Videos were shown in a common meeting place in the village where they could be projected on a wall. Each video was shown at least one time, but SHGs were free to request a second viewing during the time that the video was first shown or at a later meeting. SHGs had a total of three DG meetings. VRPs were instructed to pause the videos and read any text that appeared on the screen, after which they would conduct a question and answer session. The treatment arms contained no difference in information content, material transfers, or approximate frequency of SHG meetings compared with the control.

In the nested subtreatments, in addition to the core information about SRI, the DG videos also provided information on two additional topics perceived as potential reasons for low SRI adoption: uncertainty regarding labor costs, and farmer uncertainty in being able to perform the SRI tasks. Appendix B includes the scripts used for these messages in the videos. One of the major deterrents to adopting SRI is the potential need for extra labor, given SRI’s more involved pre-planting and planting stages. As a result, in some cases SRI results in a reallocation of labor from off-farm to farm labor, or the need for hired labor (Takahashi, 2013, Berkhout et al., 2015), but in other cases SRI does not appear to require more labor but does require additional attention to all of the particular tasks involved (Ches and Yamaji, 2015). In the SRI method prepared and presented by DG’s videos, SRI implementation required an additional day of labor per katha^13^ at each stage for an average total of 4 more days than conventional rice cultivation. The video clips exposit the potential returns to SRI, and thus, it is up to each farmer to determine if those returns would be profitable for them based on their labor availability or cost of hiring labor. On average, an additional day of female labor costs in a range of Rps 125 to 170 and male labor costs in the range of Rps 160 to 180. Thus, if SRI yields 50 kg more of rice per katha, and the government price for rice per kg is approximately Rps 10, then SRI can return Rps 300 more per katha on average.

Apart from the economic constraints to adoption there are also psychological aspects to behavior change. Very often development schemes focus on alleviating external constraints – income, credit, risk, time inconsistencies, etc.- but perceptions of one’s own abilities can affect take-up even if the economics are in one’s favor. Farmers’ outcomes depend on their own abilities to implement a new technique and the belief ex-ante that they can successfully complete it. One way in which farmers develop this belief is by watching successful peers. The peer-effects’ literature is rich with examples of how farmers learn from those who are similar to them (Conley and Udry, 2010, Bandiera and Rasul, 2006, Bursztyn et al., 2014). But peer-learning can be a slow process, like if a crop is new and there are few early adopters. Potential means to expedite this learning process is by pairing peer farmers or by incorporating them into the extension training process (Vasilaky and Leonard, 2018, BenYishay and Mobarak, 2018, Kondylis et al., 2017). This approach, however, requires at least one season to realize an experience, and that at least one farmer adopts the practice without prior experience during that season.

Thus, without sufficient examples of successful attempts by farmers like themselves, one’s own self-efficacy becomes all the more important. Bandura (1977) is a seminal work that outlines why personal self-efficacy matters for human agency. This work highlights how beliefs in one’s own abilities to produce certain outcomes and circumvent others is essential for taking action. Compte and Postlewaite (2004) present a theoretical economic model wherein a person’s history of successes and failures and the subsequent processing of this information impacts future performance. Added confidence in one’s own ability can improve future performance.

A handful of studies have investigated the role of self-efficacy in agriculture and adoption. Wuepper and Lybbert (2017) demonstrates the need to address self-efficacy in development studies, and they provide a broad overview of studies working on self-efficacy. Wuepper et al. (2020) uses an instrumental variables approach to show that farmers in Ghana with increased self-efficacy are better able to respond to periods of insufficient rainfall, and also are more likely to adopt a climate-smart technology. Kreft et al. (2021) show that self-efficacy is positively associated with the adoption of greenhouse gases mitigation measures on their farm. Niles et al. (2016) find that self-efficacy is an important predictor of whether farmers adopt climate-smart behaviors and that other measures such as attitudes and beliefs regarding climate-smart behaviors do not.

We wrote the self-efficacy message in the video based on its conceptualization in Bandura et al. (1999). First, self-efficacy is a domain-specific belief about one’s capabilities as opposed to overall self-worth. The main sources of perceived self-efficacy are one’s family and one’s peers. Second, the way in which self-efficacy is formed is via experience, observed experience of peers and persuasion. We used the latter information to develop our self-efficacy message. First, it is delivered by a farmer from the district who is from a similar social and economic status to her viewers. That farmer then describes her experience with SRI, how she started applying SRI to a sample part of a plot at first and then increased his/her coverage each year; in what way she felt supported in her adoption of the technique by Village Representative Persons (VRPs), and her potential success with the technique. Thus, the message provides an observed experience of a peer with some persuasion.

## 4 Empirical model

In Appendix A, Table A.1, we present a balance table, comparing observable inputs and outputs of the paddy production process between treatment and control groups for the baseline. As mentioned earlier, the baseline suffered from attrition, and, as a result, is a much smaller sample than the targeted sample, as shown in Fig. 2. We first test whether the observable covariates for the baseline sample are statistically similar across treatment groups. The last column presents the p-value from an F-test testing the equality of means across assignments. Overall, we can see that treatment and control are balanced along the outcomes that we will be studying: adoption, expenditures, output and profits. In Appendix A, Table A.2, we also present summary statistics from the first follow-up survey for relatively constant aspects of the household, including size of the household and details regarding the home and its assets, and see no imbalances along these dimensions. We also include the summary statistics of these variables using baseline data as point of comparison. Although, baseline data are biased by the attrition that occurred.

We estimate the following specification to capture intent-to-treat effects of the intervention in Tables 1, 2, 6, A.3, and A.4. In Panels A throughout, we estimate the treatment effect of receiving any of the DG treatment arms compared to only receiving standard extension training. Second, in Panels B throughout, we estimate the causal ITT effects for each of the four DG sub-treatments. $Y_{i}$ is the outcome variable for person i, $T_{ki}$ is treatment k for person i, where the omitted category is the control group. $T_{1}=\mathrm{DG}\;\mathrm{only}$, captures whether the household received DG training with no additional messaging; $T_{2}=\mathrm{DG}+\mathrm{labor}$ indicates whether the household received the added labor messages; $T_{3}=\mathrm{DG}+\mathrm{self}\text{-}\mathrm{efficacy}$ indicates whether the household received the added self-efficacy messages, and $T_{4}=\mathrm{DG}+\mathrm{both}$ indicates whether the household received both the labor and self-efficacy messages. The coefficients, therefore, indicate the effect of the DG treatment arm and NRLM compared to only NRLM in the control. ϵ is the error term, and we cluster our standard errors at the unit of randomization: the village.

(1) $$Y_{i}=\alpha +\beta_{1}\left(T_{1i}+T_{2i}+T_{3i}+T_{4i}\right)+\beta_{2}T_{2i}+\beta_{3}T_{3i}+\beta_{4}T_{4i}+\epsilon_{i}$$

Thus, $\beta_{1}$ captures the marginal effect of $T_{1}$, that is, DG without any additional messaging. The marginal effect of $T_{2}$, is $\beta_{1}$ + $\beta_{2}$, thus, $\beta_{2}$ alone is the marginal effect of $T_{2}$ relative to $T_{1}$. $\beta_{2}$ tells us whether the additional labor messaging increased or decreased the overall effect of DG. The same is true for $\beta_{3}$, and $\beta_{4}$.

**Table 1:** ITT treatment effects on farm outcomes.

VARIABLES	Adoption	Labor	Expenditures	Output	Profits
	Index	Days	(Rps)	(kgs)	(Rps)
*Panel A: Year One, Any DG*	(1)	(2)	(3)	(4)	(5)

Any DG	−0.00	3.83	−130	124	1,953
	(0.98)	(0.28)	(0.79)	(0.07)	(0.16)
	[0.98]	[0.47]	[0.98]	[0.35]	[0.4]

*Panel B: Year One, DG by Sub-Treatment*					

DG no messaging	−0.02	4.82	−539	255	5,184
	(0.76)	(0.37)	(0.41)	(0.04)	(0.02)
	[0.87]	[0.74]	[0.75]	[0.20]	[0.20]
DG + labor	0.02	1.16	984	−170	−5,105
	(0.84)	(0.87)	(0.24)	(0.25)	(0.04)
	[0.87]	[0.87]	[0.63]	[0.63]	[0.20]
DG + self-efficacy	0.03	−7.22	294	−248	−5,210
	(0.71)	(0.23)	(0.70)	(0.07)	(0.04)
	[0.87]	[0.63]	[0.87]	[0.28]	[0.20]
DG + both	0.03	1.71	340	−111	−2,608
	(0.69)	(0.81)	(0.64)	(0.46)	(0.33)
	[0.87]	[0.87]	[0.87]	[0.77]	[0.73]
Observations	2,156	2,156	2,156	2,156	2,156
Control Group Mean, Year One	1.054	41.28	6,967	635	6,793
Control Group Trend	−0.34		−3191	30.88	

*Panel C: Year Two, Any DG*	(1)${}^{‡}$	(2)	(3)	(4)	(5)

Any DG		1.42	−1,450	88.5	2,851
		(0.75)	(0.44)	(0.39)	(0.33)
		[0.75]	[0.59]	[0.59]	[0.59]

*Panel D: Year Two, DG by Sub-Treatment*					

DG no messaging		1.77	−1,301	106	3,823
		(0.80)	(0.50)	(0.54)	(0.34)
		[0.96]	[0.96]	[0.96]	[0.96]
DG + labor		0.90	420	87.8	947.48
		(0.91)	(0.51)	(0.66)	(0.82)
		[0.96]	[0.96]	[0.96]	[0.96]
DG + self-efficacy		−2.32	−471	−26	−1,968
		(0.77)	(0.40)	(0.89)	(0.57)
		[0.96]	[0.96]	[0.96]	[0.96]
DG + both		0.02	−501	−119	−2,650
		(1.00)	(0.34)	(0.50)	(0.45)
		[0.96]	[0.96]	[0.96]	[0.96]
Observations		2,061	2,061	2,061	2,061
Control Group Mean, Year Two		49.61	5,722	747	8,666

Standard errors, clustered at the village level, p-values reported in parentheses and False Discovery Rate (FDR) pvalues reported in brackets Anderson (2008). All variables are measured per farm. The effects of DG + labor, DG + self-efficacy and DG + both are the marginal effects relative to DG no messaging.**Adoption Index** is an index ranging from 0–5, which reflects whether the farmer implemented the practices broken down in Table A.3 according to SRI principles. **Labor Days** represents the total labor days (both family and hired labor) on cleaning, pesticide, threshing, and harvesting, which are the activities for which we have responses in both Year One and Year Two. **Expenditures** represents the common set of total expenditures between Year One and Year Two spent on inputs including fertilizer, machine rental and irrigation. In Year Two we did not conduct a midline survey, thus **Expenditures** reflect endline (harvest) costs only. **Output** is total kilograms of rice produced on the farm. Estimated Profit is a constructed variable and is equal to harvest storedx$p_{r}$ + harvest soldx$p_{f}$ - **Expenditures** - **Hired Labor**, where $p_{f}$ is the reported farm gate price at which a farmer sold her rice, and $p_{r}$ is the estimated retail price each year (25.6 Rps/kgs and 27.8 Rps/kgs from Bhoi et al. (2019)). For the full set of expenditures surveyed by year see Table 2. Control Group Trend is Year One’s control group mean outcome minus the Baseline’s control group mean outcome. We control for whether a household’s total self-reported farm output aligns with the aggregate of their reported sold, consumed, stored, and shared paddy within a margin of error of 100 kilograms throughout. ${}^{‡}$Questions about general adoption practices were not asked in the final endline.

**Table 2:** ITT treatment effects on input components per farm (Rps).

	Inputs					Hired Labor Expenses								
	SRI Relevant			All other		SRI Relevant			All other					
VARIABLES	Seed	Irrigation	Fertilizer	Machinery	Livestock	Land prep	Transplantation	Weeding	Nursery	Water pumping	Cleaning	Pesticide	Threshing	Harvesting
*Panel A: Year One, Any DG*	(1)	(2)	(3)	(4)	(5)	(6)	(7)	(8)	(9)	(10)	(11)	(12)	(13)	(14)
Any DG	−8.66	−26.08	−84.93	−19.90	0.14	−85.06	138.94	−189.77	1.71	−9.92	29.64	−0.11	119.03	92.32
	(0.92)	(0.82)	(0.71)	(0.95)	(0.99)	(0.43)	(0.53)	(0.26)	(0.94)	(0.75)	(0.41)	(0.99)	(0.27)	(0.61)

*Panel B: Year One, DG by Sub-Treatment*														

DG no messaging	−17.17	−51.10	−158.26	−329.92	−18.70	−130.93	−262.49	−146.97	−19.39	−16.00	49.54	15.11	60.94	126.03
	(0.89)	(0.74)	(0.55)	(0.37)	(0.27)	(0.34)	(0.23)	(0.58)	(0.43)	(0.69)	(0.29)	(0.53)	(0.66)	(0.67)
DG + labor	−31.91	301.81	−16.93	699.41	15.19	181.42	552.39	−135.47	11.46	−27.87	−62.05	−34.39	110.33	123.30
	(0.80)	(0.19)	(0.94)	(0.17)	(0.46)	(0.26)	(0.04)	(0.57)	(0.66)	(0.48)	(0.22)	(0.13)	(0.57)	(0.76)
DG + self-efficacy	−27.28	−87.49	7.51	374.50	5.12	104.46	735.31	−3.91	50.77	51.70	−27.45	2.50	9.05	−221.15
	(0.85)	(0.60)	(0.98)	(0.45)	(0.75)	(0.48)	(0.23)	(0.99)	(0.40)	(0.43)	(0.63)	(0.93)	(0.95)	(0.49)
DG + both	86.07	−109.95	282.79	167.32	51.54	−92.13	321.66	−29.69	22.44	2.31	8.35	−26.95	105.63	−42.36
	(0.54)	(0.50)	(0.40)	(0.65)	(0.04)	(0.42)	(0.20)	(0.90)	(0.45)	(0.96)	(0.91)	(0.23)	(0.64)	(0.90)
Observations	2,156	2,156	2,156	2,156	2,156	2,156	2,156	2,156	2,156	2,156	2,156	2,156	2,156	2,156
Control Group Mean, Year One	1,035	1,458	1,742	3,767	53.68	632.4	1,847	1,268	141.8	147	167.9	103.8	497.9	1,010
Control Group Mean, Baseline	1,068	3,141	3,869	3,182	38.64	295.19	540.56	409.53	122.59	98.30		89.78		630
Control Group Trend pvalue	0.83	0.00	0.00	0.13	0.44	0.04	0.00	0.00	0.51	0.29		0.62		0.07

*Panel C: Year Two, Any DG*	(1)${}^{‡}$	(2)	(3)	(4)	(5)${}^{‡}$	(6)${}^{‡}$	(7)${}^{‡}$	(8)${}^{‡}$	(9)${}^{‡}$	(10)${}^{‡}$	(11)	(12)	(13)	(14)

Any DG		77.66	−1,613	84.91							2.55	11.18	65.02	310.84
		(0.67)	(0.37)	(0.04)							(0.94)	(0.62)	(0.43)	(0.18)

*Panel D: Year Two, DG by Sub-Treatment*														

DG no messaging		−2.58	−1,373	117.02							25.36	61.05	64.83	368.04
		(0.99)	(0.45)	(0.13)							(0.61)	(0.11)	(0.62)	(0.41)
DG + labor		581.18	−127.87	−59.69							15.30	−41.12	88.53	−123.92
		(0.09)	(0.68)	(0.50)							(0.80)	(0.36)	(0.62)	(0.81)
DG + self-efficacy		−131.98	−346.86	−19.65							−31.98	−72.93	−25.71	−232.77
		(0.63)	(0.24)	(0.85)							(0.58)	(0.06)	(0.86)	(0.64)
DG + both		−74.16	−411.68	−42.62							−66.27	−78.10	−47.01	131.97
		(0.76)	(0.17)	(0.66)							(0.17)	(0.06)	(0.75)	(0.81)
Observations		2,061	2,061	2,061							2,061	2,061	2,061	2,061
Control Group Mean, Year Two		2048	3411	263.9							369	164.3	138.8	1182

Standard errors, clustered at the village level, p-values reported in parentheses. The effects of DG + labor, DG + self-efficacy and DG + both are the marginal effects relative to DG no messaging. **Inputs** is the amount spent in Indian Rupees on each input on the farm in a season, and **Hired Labor Expenses** is the amount spent in Indian Rupees on hiring labor in a season on a specific practice. **SRI Relevant** refers to practices where we would expect differences in inputs between traditional and SRI grown rice. ${}^{‡}$ In Year Two we did not conduct a midline survey, thus, we did not survey farmers on their midline practices. Control Group Trend is Year One’s control group mean outcome minus the Baseline’s control group mean outcome, and the pvalue in parentheses reflects an unpaired ttest between the Baseline and Year One control group means. We control for whether a household’s total self-reported farm output aligns with the aggregate of their reported sold, consumed, stored, and shared paddy within a margin of error of 100 kilograms throughout.

### 4.1 Average treatment effects

We first focus on the effects of the intervention on five outcomes as outlined in our pre-registration: adoption, labor use, expenditures on farm inputs, output and estimated profits.^14^ While the messages in the intervention were posed at a per acre basis we are also interested in the practical impacts of the intervention for the average farmer in levels and thus report our results in Table 1 in levels, and per acre in Table 6 for Year One.

Results are reported for Any DG in Panels A and C for Year One and Two respectively, and then the intervention is broken down by DG without messaging followed by DG with a labor message, DG with a self-efficacy message, and DG with both labor and self-efficacy messages in Panels B and D. Standard errors are clustered at the village level with p-values reported in parentheses and False Discovery Rate p-values (Anderson, 2008), which correct for multiple hypothesis testing (MHT), are reported in brackets. In addition to the reported p-values, we discuss the average change in each outcome as well as possible values for the true average outcome that are compatible with our data and model within a 95% CI to highlight the economic effect of the treatment, and the uncertainty around that effect (without corrections for MHT).

Column 1 of Table 1, Table 6 report on an adoption index, constructed from questions regarding rice growing practices. The specific questions on SRI practices and their answers are included in Appendix A, Table A.3, including the number of weedings in a season, the age of saplings before transplantation, the number of saplings to be transplanted, spacing between seedlings and spacing between rows. If the respondent answered the question within the range of what is required for SRI practices, as taught by Digital Green, she received a 1 on that question and this was summed for the final index, which ranges from 0 to 5. We observe zero average change in rice practices between the treated and control groups in Year One with a possible range of values from −0.11 to 0.11 (95% CI). That is, at least among these practices, farmers do not report behaving differently when exposed to DG videos as compared to standard extension training.

Columns 2 and 3 of Table 1 report changes in intermediate outcomes in levels (and Table 6 per acres), for labor days and expenditures in growing rice. Note that in Year Two we did not conduct a midline survey, where we captured data on mid-season practices; thus, in order to compare the two years we report only the common set of endline practices including cleaning, pesticide, threshing, and harvesting. Column 2 indicates an average change in labor days of 3.38 (−9.30 days/acre) for Any DG in Panel A, with possible values between −3 and 11 days (−50 and 30 days/acre) (95% CI). Column 3 indicates an average change in expenditures −130 Indian Rupees (298 Rps/acre) for Any DG in Panel A, with possible values between −1,090 and 828 Rupees (−3,739 and 4,335 Rps/acre), approximately −16 and 12 USD (−28 and 32 USD/acre) (95% CI). Thus, unlike the zero average effect of the interventions on the adoption index, we observe non-zero average effects on inputs, albeit imprecisely measured, which are not statistically significant with and without MHT corrections.

Columns 4 and 5 report the effects of the intervention on final outcomes. For Any DG, output increased by 124 kilograms per farm (250 kgs/acre) for Any DG in Panel A, with possible values between −8 and 255 kilograms per farm (−56 and 566 kgs/acre) (95% CI). This effect on output is economically significant, as mean output per farm was 635 kilograms per farm (1250 kgs/acre), a nearly 19% (20%) increase. When broken down by treatment arm, the DG no messaging arm and DG + both arm experienced the greatest average changes 255 (range of 10 to 500 kilograms per farm) and 144 kilograms per farm (range of −70 to 359 kilograms per farm), and 374 (range of −149 to 898 kilograms per acre) and 516 (range of −70 to 1103 kilograms per acre) per acre.

Estimated Profit is a constructed variable and is equal to the amount of output that the farmer stored times the retail price of rice at the time, plus the amount of harvest sold times the self-reported farm gate price at which it was sold. Since we did not survey farmers on retail prices for rice we used the estimated retail price in Year One and Two (25.6 Rps/kgs and 27.8 Rps/kgs) taken from Bhoi et al. (2019). Estimated profits per farm experienced an average change of 1,953 Rupees (3,335 Rps/acre) for Any DG in Panel A, with possible values between −753 and 4658 Rupees (−2,848 and 9,519) (95% CI). When broken down by subtreatment arms, the average change for DG no messaging was 5184 Rupees per farm (7,215 Rps/acre), with possible values between 717 and 9650 Rupees (−254 and 14,683 Rps/acre) (95% CI).

Taken together, many of the latter effects exhibit wide confidence intervals and do not meet the criteria for statistical significance once multiple hypotheses testing, using the False Discovery Rate, is applied.

**Fig. 3:**
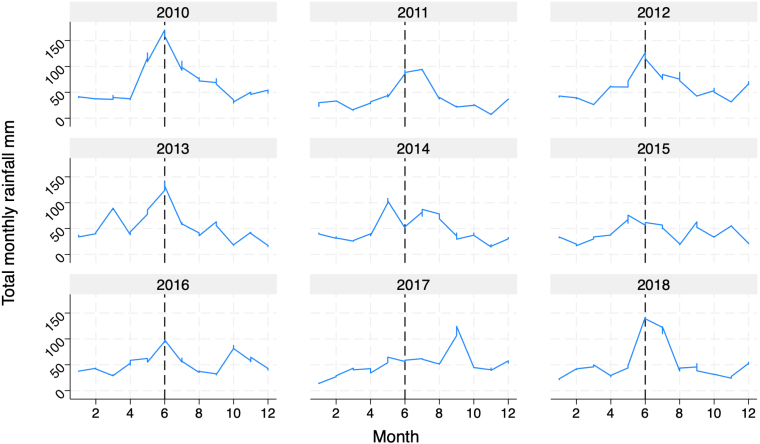
Total monthly rainfall by year study years = 2014 and 2015.

Climate hazards group infraRed precipitation with station data.

Turning to Year Two effects, the direction of the effects is largely the same as in Year One, but the magnitude of the effect sizes is, in most cases, reduced. We see that the average effect on labor days is less than half of Year One’s effects, while expenditures declined by more than twice the Year One’s effects. The average effect on output is reduced for Any DG in Panel A and for DG no messaging in Panel B, which is the treatment arm that exhibited the greatest economic impact in Year One. One possibility for this is that farmers perceive SRI to be more effective when precipitation is high, and Year Two (2015) had lower rainfall relative to Year One (2014) as shown in Fig. 3. Bezabih et al. (2016) shows that higher temperatures and reduced rainfall can lead to farmers being less likely to adopt SRI. Another possibility is that farmers disadopted some of the practices that they acquired in Year One. Barrett et al. (2021) shows that disadoption of SRI is quite common, even when first year yield and profit gains are statistically and economically significant. They suggest that disadoption could be the result of heterogeneous returns to SRI, although they cannot point to any observable source of such heterogeneity. All that being said, with diminished effect sizes and equally imprecise estimates as Year One, the effects on our five outcomes are not statistically significant at the 5% level with and without MHT corrections.

**Fig. 4:**
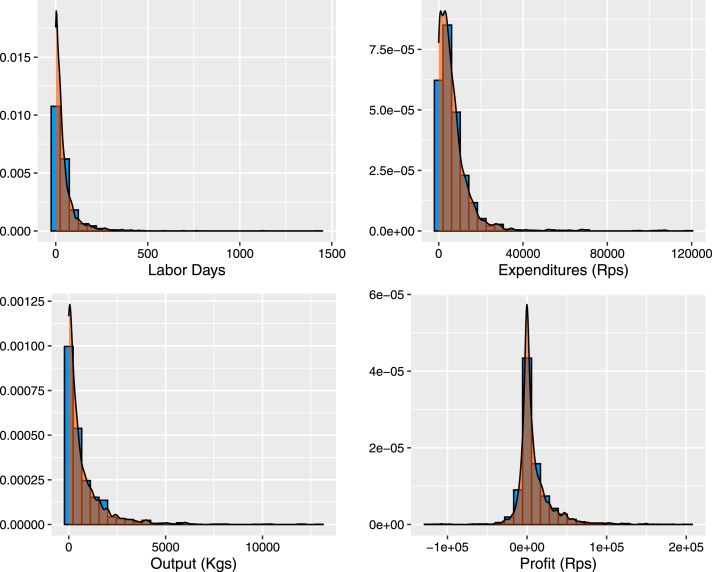
Distribution of outcome variables. Notes: Each plot represents a histogram of our outcomes in year one (excluding the adoption index) with a kernel density plot overlaid.

## 5 Quantile treatment effects

The above analysis presents estimated effects that are large, but after applying MHT corrections, are not statistically significant in levels or per acre. However, with the exception of the adoption index, all of our outcome variables exhibit distributions with fat tails, which can make an OLS specification inappropriate. Fig. 4 depicts histograms of the data for the five outcome variables with substantial right tails. The data generating process of our outcomes puts the classical inference that we used above into question, and inferences are likely to be wrong. In particular, the normality parameter v, which reflects the degrees of freedom in t-distribution, dictates whether the variance of the Student t-distribution is finite. A normality parameter v ∈ (1,2] refers to an infinite variance for the Student t (Kruschke, 2015). With the exception of the adoption index, which ranges between 0 and 5 by construction, the remaining four outcome variables fit this criterion according to Table 3. This would render the classical confidence intervals for labor, output and profits to be moot.^15^

The issue of fat tails has been studied in several contexts, from development economics to finance and advertising, each for different reasons. In advertising, fat tails occur in outcomes such as clicks or purchases because there are generally few successes among hundreds of ad campaigns leading to long right tails. However, firms are interested in detecting small increases in revenue despite large standard errors of revenue itself (Lewis and Rao, 2015). In finance, there is evidence that fat tails in daily exchange rate and equity indices can have important effects on volatility estimates and forecasts (Jacquier et al., 2004). Similarly, the adoption of new technologies in developing countries as an outcome may follow a long right tail in which there are few adopters and the bulk of observations are zeros. Deaton and Cartwright (2018) warns that these asymmetric distributions of treatment effects pose threats to significance testing. Meager (2019) investigates the returns to offering microcredit across seven different studies, in which the bulk of the returns accumulate to top income (75th percentile of income) individuals.

**Table 3:** Estimated normality parameter by outcome.

	Mode	95% HDI	
Adoption Index	123	60	240
Labor Days	1.46	1.33	1.60
Expenditures (Rps)	2.26	2.03	2.54
Output (kgs)	1.24	1.13	1.36
Profits (Rps)	1.30	1.18	1.45

The Mode represents the mode of the posterior distribution for the normality parameter from estimating the treatment effect of Any DG versus the Control in the Bayesian hierarchical model for each respective outcome: Adoption Index, Labor Days, Expenditures (Rps), Output (kgs), and Profits (Rps).The 95% Highest Density Interval (HDI) lists the most likely estimated parameter values that comprise 95% of the distribution of possible effects with HDIlo and HDIhigh as the bounds.

**Fig. 5:**
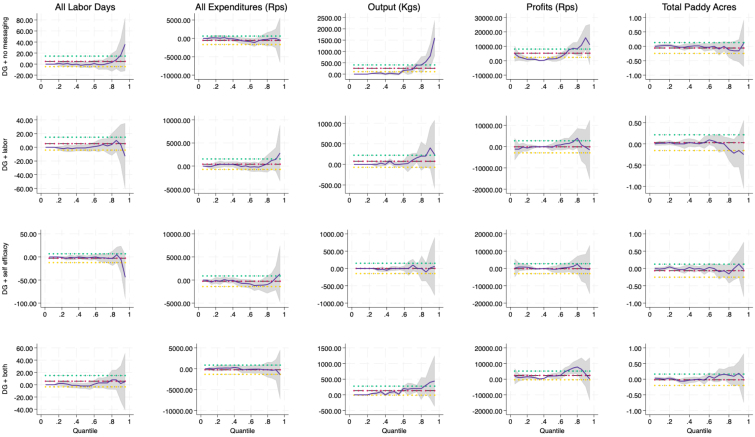
Quantile treatment effects of DG. Notes: Each plot represents the treatment effect (blue line) for each treatment arm on the outcomes using a quantile regression with the treatment effect size on the y-axis and each quantile (estimated at quantile intervals of 0.05) along the x-axis. The gray area represents 95% confidence, and the red, blue and yellow lines represent least squares estimate and its confidence interval. (For interpretation of the references to color in this figure legend, the reader is referred to the web version of this article.)

A common next step to estimating average treatment effects in the presence of fat tails is to estimate quantile treatment effects. Quantile treatment effects estimators offer a way to explore how treatment effects vary within the population. In particular, we would expect the imprecision to occur in the right tail of our outcome distributions, since the left tail is censored at zero for inputs and outputs. Fig. 5, Fig. 9 provide a visual depiction of the quantile treatment effects across all outcomes, while Table 4 hones in on output and yield alone. Fig. 5 provides a clear indication that there is both higher impacts of the intervention in the upper quantiles of the outcomes in levels as well as greater imprecision of the estimated impact in the upper tails. Fig. 9, which presents the impacts on outcomes per acre, indicates that the larger effect sizes in the upper tails are tempered when the outcomes are scaled by acres, suggesting that larger farmers can potentially benefit more than smaller farmers from added DG messaging. However, once we control for farm size, the messaging does not seem to add any additional benefit beyond the main DG videos. What does not change is that the estimates are still more imprecisely measured in the upper quantiles.

**Table 4:** OLS, Quantile regressions, and Weighted quantile treatment effects in year one.

	AnyDG	DG	DG +	DG +	DG +
		no messaging	labor	self-efficacy	both
*Output* (kgs) *controlling for number of paddy acres*					

ATE	113.34	263.4	69.99	7.09	114.53
	(0.081)	(0.028)	(0.475)	(0.928)	(0.282)
Quantile .25	0	20.92	0	0	32.42
	(1)	(.208)	(1)	(1)	(0.057)
Quantile .5	44.27	73.03	46.63	−22.25	73.37
	(0.057)	(0.065)	(0.171)	(0.42)	(0.043)
Quantile .75	108.7	171.56	72.04	65.88	127.39
	(0.005)	(0.026)	(0.171)	(0.277)	(0.042)

*Output* (kgs) *NOT controlling for number of paddy acres*					

ATE	117.23	257.86	75.44	2.75	132.8
	(0.085)	(0.042)	(0.458)	(0.974)	(0.232)
Quantile .25	5	30	0	0	40
	(0.668)	(0.072)	(1)	(1)	(0.017)
Quantile .5	20	50	20	0	100
	(0.469)	(0.357)	(0.599)	(1)	(0.037)
Quantile .75	200	280	160	0	200
	(0.005)	(0.125)	(0.199)	(1)	(0.144)
WAQ	13.82	19.34	13.73	−8.02	36.31
	(0.46)	(0.48)	(0.61)	(0.77)	(0.16)
Mean BP	30.40	30.4	27.40	−2.53	75.3
	[−9.43–70]	[−8.48–70]	[−28–82.7]	[−60.5–53.9]	[13.5–138]

*Yield* (kgs/acres *for those who planted*)					

ATE	237.98	379.87	47.85	−3.16	493.15
	(0.141)	(0.156)	(0.846)	(0.986)	(0.1)
Quantile .25	66.47	144.7	22.03	−38.16	108.12
	(0.019)	(0.095)	(0.594)	(0.384)	(0.008)
Quantile .5	165.57	276.89	148.95	−0.66	227.42
	(0.009)	(0.005)	(0.08)	(0.994)	(0.008)
Quantile .75	111.63	210.09	−88.37	111.63	111.63
	(0.121)	(0.024)	(0.285)	(0.34)	(0.349)
WAQ	58.58	100.27	22.03	2.32	82.78
	(0.11)	(0.06)	(0.67)	(0.96)	(0.10)
Mean BP	156	228	75.6	0.725	156
	[34–278]	[96.1–361]	[−44.9–196]	[−128–133]	[35.8–279]

*IHS(Output)*					

ATE	0.15	0.32	0.04	−0.14	0.37
	(0.504)	(0.322)	(0.899)	(0.676)	(0.253)
Quantile .25	2.31	4.09	0	0	4.38
	(0.552)	(0)	(1)	(1)	(0)
Quantile .5	0.06	0.06	0	0	0.29
	(0.486)	(0.658)	(1)	(1)	(0.032)
Quantile .75	0.22	0.3	0.18	0	0.22
	(0.015)	(0.028)	(0.196)	(1)	(0.078)
WAQ	0.15	0.28	0.15	0.05	0.27
	(0.02)	(0.002)	(0.11)	(0.57)	(0.002)

*IHS(Yield)*					

ATE	0.18	0.4	0.01	−0.03	0.33
	(0.454)	(0.234)	(0.988)	(0.93)	(0.328)
Quantile .25	0.47	0.83	0.18	−0.42	0.68
	(0.237)	(0.133)	(0.76)	(0.8)	(0.169)
Quantile .5	0.22	0.34	0.2	−0.01	0.29
	(0.015)	(0.003)	(0.093)	(0.928)	(0.009)
Quantile .75	0.07	0.13	−0.06	0.07	0.07
	(0.173)	(0.051)	(0.402)	(0.376)	(0.326)
WAQ	0.14	0.27	0.03	0.01	0.23
	(0.02)	(0.002)	(.75)	(0.88)	(0.009)

Standard errors, clustered at the village level for average treatment effect (ATE) in OLS estimation. p-values reported in parentheses. Mean BP refers to the Mean of the Bayesian Posterior Distribution of the treatment effect estimator. The 95% Highest Density Interval (HDI) is reported in brackets for Mean BP and lists the most likely estimated parameter values that comprise 95% of the distribution of possible effects with HDIlo and HDIhigh as the bounds. **IHS** refers to inverse hyperbolic sine function. Regressions are clustered at the village level, p-values reported in parentheses. **WAQ** stands for weighted average quantile (waq) estimator as detailed in Athey et al. (2023). This table does not control for *share* as in Table 1, Table 6.

In Table 4, we compare the effects across several different methodologies: the average treatment effect, quantile effects, and the weighted average quantile (WAQ) effect (Athey et al., 2023) for several different variations of the main outcome of interest: output, yield, and transformations of output as well as yield by the inverse hyperbolic sine (IHS) transformation. Note that the sub-treatment effects in this table are *not* relative to the main DG only arm, but rather, are estimated as mutually exclusive treatments. This is for ease of comparison between the treatment effects across the various methods. The WAQ effect estimator (WAQ) allows for aggregation of treatment effects where the weights can differ across quantiles (Athey et al., 2023). The IHS transformation is used to transform right-skewed variables, which may include zero or negative values (Bellemare and Wichman, 2020, Aihounton and Henningsen, 2021). In our case, both yield and output contain zero values. Because of interpretation issues (Aihounton and Henningsen, 2021, Chen and Roth, 2023), we do not transform the coefficients or interpret the magnitude of the treatment effects. Overall, the results show that the DG + both treatment arms exhibit economically and statistically significant effects on output and transformed output for the 25% and 50% quantiles, a 6%–16% increase over baseline, and the DG without messaging as well as DG + both treatment arms consistently exhibit economically and statistically significant effects on yield and transformed yield for the 50% quantiles, a 25%–31% increase over baseline. The WAQ estimator confirms that the observations above the median are particularly noisy and puts little weight on the effects in the upper tails to estimate a more precise average treatment effect. Fig. 8 depicts the weights for the WAQ treatment effect on output per farm and per acre. The WAQ estimates in Table 4 largely confirm that the first and last arms are statistically significant, particularly for the transformed outcomes (although not for output in levels, which exhibits more dispersion than yields).

## 6 Bayesian program effects

A Bayesian approach does not foundationally rely on assuming a thin-tailed data generating process in order to generate asymptotic normality of estimators. Rather, the researcher can specify an appropriate generating process for the data at hand, combined with a set of priors on the parameters that govern this data generating process. Another benefit of a Bayesian analysis is that it produces a posterior distribution of the average treatment effects rather than a single point estimate of the treatment’s effect. This can be a more informed manner of studying interventions that are applied to fat tailed data, when treatment effects exhibit wide confidence intervals in a frequentist design.

In this section, we revisit the estimation of our average treatment effects using a Bayesian hierarchical framework and compare the results found in the previous sections. Such a comparison allows us to highlight the differences between approaches. Given that we did not specify priors before the study took place we use two different sets of priors to describe our data. The first set, as suggested by Kruschke (2013), are normal priors with large standard deviation for μ, broad uniform priors for σ, and a shifted-exponential prior for the normality parameter ν. The latter spreads “prior credibility fairly evenly over nearly normal and heavy tailed data” Kruschke (2013). We use a t-distribution to model our sample, and generate vectors of random draws from the posterior distribution of the center (μ) and spread or scale (σ) of the distribution, as well as a measure of normality (ν). The normality parameter, ν, corresponds to the degrees of freedom of the t-distribution in the context of sampling distributions (Kruschke, 2013). The procedure uses a Bayesian MCMC process implemented in JAGS (Plummer 2003).^16^ The second set of priors are more conservative, with much smaller variance than the first.

Let M and S be the mean and the standard deviation of the pooled data. The implementation then follows Meredith and Kruschke (2021). For k=0 (control), 1 (treatment), the ith individual’s outcome y is given by:

(2) $$y_{k,i}=\mu_{k}+\sigma_{k}*t_{k,i},where$$

$$t_{k,i}∼t\left(v\right)$$

$$\mu_{k}∼N\left(M,1000*S\right)$$

$$\sigma_{k}∼U\left[S/1000,S*1000\right]$$

ν−1∼Exp(1/29),ν>=1

Table 5, Table 8 report results from the above priors of 100,000 draws from the posterior distribution of the average treatment effect, $\mu_{1}-\mu_{0}$ for each arm on each outcome variable. Note that, as is the case with Table 4, the sub-treatment effects reported in Table 5, Table 8 estimate mutually exclusive (and *not* relative) effects of the sub-treatment arms. The 95% Highest Density Interval (HDI) indicates the most likely estimated parameter values that comprise 95% of the distribution of possible effects. The Prob < 0 or > 0 is the probability that the true effect is less than or greater than zero. Note that the treatment arms are mutually exclusive in this estimation, unlike in Section 4.1, in which the subtreatment effects were estimated relative to the main treatment arm. The second set of priors that we apply are more conservative and differ in that the variance of the mean and the standard deviation is 5*S, and ν∼Gamma(30,30),ν>=1. The main findings are unchanged from the results described here with broad priors and we report the results from the second set of priors in Table A.5, Table A.6.

Beginning with adoption, as with the average treatment effect estimator, zero is the most likely effect of the DG across all arms on adoption. Looking at inputs and outputs, as with the quantile regressions, the main arm without messaging and the DG + both arm produce the most favorable results in terms of output (yield), 30.4 kilograms per farm (228 kgs/acre) and 75 kilograms per farm (156 kgs/acre) increase compared to standard training, respectively. The probability that the true value is greater than zero ranges from 94%–100%. Profits are also highest for these treatment arms. In most cases expenditures and labor days also declined, except for labor days when looking at yields. Thus, compared to the control group of SRI alone, DG led to increases in productivity and zero is not the most likely effect of the main DG treatment or the DG treatment with both messages. Overall, the mean of the Bayesian effect sizes are smaller than the initial average treatment effect estimates. Interestingly, the mean of the Bayesian posterior for output under DG + both, 75 kilograms per farm, lies in between the 25% (40 kgs) and 50% quantile (100 kgs) regressions effects for output. Similarly, the mean of the Bayesian posterior for yield, 228 kilograms per acre for DG without messaging and 156 kilograms per acre for DG + both, lies in between the 25% and 50% quantile (100 kgs) regressions for yields.

**Table 5:** Mean Bayesian posterior distribution of treatment effects.

		Effect	HDIlo	HDIhigh	Prob <0	Prob >0
*Adoption Index*						

	DG no messaging	−0.003	−0.08	0.07	0.53	0.47
	DG + labor	−0.008	−0.13	0.11	0.55	0.45
	DG + self_efficacy	0.008	−0.11	0.13	0.44	0.56
	DG + both	0.008	−0.10	0.12	0.44	0.56

*Labor Days*						

	DG no messaging	−1.32	−3.8	1.21	0.85	0.15
	DG + labor	−.97	−4.74	2.84	0.69	0.31
	DG + self_efficacy	−1.27	−4.91	2.37	0.75	0.25
	DG + both	−1.29	−4.92	2.17	0.76	0.24

*Expenditures* (Rps)						

	DG no messaging	−284	−728	166	0.89	0.11
	DG + labor	36.6	−615	672	0.46	0.54
	DG + self_efficacy	−608	−1210	0.25	0.98	0.02
	DG + both	−40.3	−664	582	0.55	0.45

*Output* (kgs)						

	DG no messaging	30.4	−8.48	70	0.06	0.94
	DG + labor	27.40	−28	82.7	0.17	0.83
	DG + self_efficacy	−2.53	−60.5	53.90	0.54	0.46
	DG + both	75.3	13.5	138	0.007	0.99

*Profits* (Rps)						

	DG no messaging	590	−193	1380	0.07	0.93
	DG + labor	262	−919	1440	0.33	0.67
	DG + self_efficacy	−175	−1230	865	0.63	0.37
	DG + both	1620	315	2870	0.005	0.99

The 95% Highest Density Interval (HDI) indicates the most likely estimated parameter values that comprise 95% of the distribution of possible effects with HDIlo and HDIhigh as the bounds. The Prob < 0 or > 0 is the probability that the true effect is less than or greater than zero.

**Table 6:** ITT treatment effects on farm outcomes (per acre).

VARIABLES	Adoption	Labor	Expenditures	Yield	Profits
	Index	Days/Acre	(Rps/Acre)	(kgs/Acre)	(Rps/Acre)
*Panel A: Year One, Any DG*	(1)	(2)	(3)	(4)	(5)

Any DG	−0.00	−9.30	298	250	3,335
	(0.98)	(0.65)	(0.88)	(0.12)	(0.29)
	[0.98]	[0.98]	[0.98]	[0.6]	[0.73]

*Panel B: Year One, DG by Sub-Treatment*					

DG no messaging	−0.02	−16.71	−1,314	375	7,215
	(0.76)	(0.42)	(0.60)	(0.16)	(0.06)
	[0.84]	[0.84]	[0.84]	[0.76]	[0.4]
DG + labor	0.02	4.31	1,221	−306	−8,181
	(0.84)	(0.80)	(0.69)	(0.35)	(0.05)
	[0.84]	[0.84]	[0.84]	[0.84]	[0.4]
DG + self-efficacy	0.03	3.15	3,040	−370	−11,382
	(0.71)	(0.82)	(0.37)	(0.19)	(0.02)
	[0.84]	[0.84]	[0.84]	[0.76]	[0.4]
DG + both	0.03	20.57	2,201	142	3,042
	(0.69)	(0.28)	(0.40)	(0.70)	(0.60)
	[0.84]	[0.84]	[0.84]	[0.84]	[0.84]
Observations	2,156	1,944	1,944	1,944	1,944
Control Group Mean, Year One	0.22	107	15 947	1250	12 207
Control Group Trend			452	-600	

*Panel C: Year Two, Any DG*	(1)${}^{‡}$	(2)	(3)	(4)	(5)

Any DG		1.99	−114	90.1	2,066
		(0.44)	(0.76)	(0.06)	(0.06)
		[0.71]	[0.76]	[0.15]	[0.15]

*Panel D: Year Two, DG by Sub-Treatment*					

DG no messaging		1.56	−129	88.2	2,299
		(0.73)	(0.76)	(0.37)	(0.25)
		[0.96]	[0.96]	[0.96]	[0.96]
DG + labor		1.75	426	56	481
		(0.72)	(0.24)	(0.61)	(0.84)
		[0.96]	[0.96]	[0.96]	[0.96]
DG + self-efficacy		0.51	−142	27.6	−343
		(0.93)	(0.70)	(0.81)	(0.88)
		[0.96]	[0.96]	[0.96]	[0.96]
DG + both		−0.48	−196	−69	−983
		(0.92)	(0.55)	(0.50)	(0.65)
		[0.96]	[0.96]	[0.96]	[0.96]
Observations		2,032	2,035	2,036	2,032
Control Group Mean, Year Two		39.1	3685	472	5240

Standard errors, clustered at the village level, p-values reported in parentheses and False Discovery Rate (FDR) pvalues reported in brackets Anderson (2008). All variables are measured per acre. The effects of DG + labor, DG + self-efficacy and DG + both are the marginal effects relative to DG no messaging. **Adoption Index** is an index ranging from 0–5, which reflects whether the farmer implemented the practices broken down in Table A.3 according to SRI principles. **Labor Days** represents the total labor days (both family and hired labor) on cleaning, pesticide, threshing, and harvesting, which are the activities for which we have responses in both Year One and Year Two. **Expenditures** represents the common set of total expenditures between Year One and Year Two spent on inputs including fertilizer, machine rental and irrigation. In Year Two we did not conduct a midline survey, thus **Expenditures** reflect endline (harvest) costs only. **Output** is total kilograms of rice produced on the farm. **Estimated Profit** is a constructed variable and is equal to harvest storedx$p_{r}$ + harvest soldx$p_{f}$ - **Expenditures** - **Hired Labor**, where $p_{f}$ is the reported farm gate price at which a farmer sold her rice, and $p_{r}$ is the estimated retail price each year (25.6 Rps/kgs and 27.8 Rps/kgs from Bhoi et al. (2019)). For the full set of expenditures surveyed by year see Table 7. Control Group Trend is Year One’s control group mean outcome minus the Baseline’s control group mean outcome. We control for whether a household’s total self-reported farm output aligns with the aggregate of their reported sold, consumed, stored, and shared paddy within a margin of error of 100 kilograms throughout. ${}^{‡}$Questions about general adoption practices were not asked in the final endline.

**Table 7:** ITT treatment effects on input components per acre (Rps).

	Inputs					Hired Labor Expenses								
	SRI Relevant			All other		SRI Relevant			All other					
VARIABLES	Seed	Irrigation	Fertilizer	Machinery	Livestock	Land prep	Transplantation	Weeding	Nursery	Water pumping	Cleaning	Pesticide	Threshing	Harvesting
*Panel A: Year One, Any DG*	(1)	(2)	(3)	(4)	(5)	(6)	(7)	(8)	(9)	(10)	(11)	(12)	(13)	(14)

AnyDG	−242.02	10.15	−3.25	177.00	−14.43	−94.84	−2,949.54	88.95	87.81	122.01	25.65	−10.29	77.10	75.56
	(0.69)	(429.40)	(597.71)	(1,112.02)	(55.34)	(276.84)	(3,587.91)	(459.07)	(94.74)	(97.17)	(68.85)	(48.71)	(217.17)	(312.40)

*Panel B: Year One, DG by Sub-Treatment*														

DG no messaging	−692.85	−373.88	−312.74	−657.42	−57.79	−214.14	−3,652.27	−141.89	3.93	127.58	16.13	−30.02	−273.83	−192.97
	(0.22)	(469.62)	(709.99)	(1,389.15)	(65.01)	(359.00)	(4,201.76)	(519.80)	(76.14)	(177.32)	(90.56)	(54.24)	(240.34)	(409.69)
DG + labor	−27.26	444.83	−480.69	1,285.09	75.67	201.75	964.21	−38.26	195.66	−142.23	−92.93	−53.16	372.90	321.26
	(0.93)	(590.93)	(712.33)	(1,791.49)	(94.12)	(381.97)	(1,582.89)	(539.66)	(204.86)	(180.63)	(91.49)	(46.06)	(298.56)	(574.02)
DG + self-efficacy	1,147.95	475.18	849.87	1,102.13	−48.46	499.34	1,293.87	904.11	148.01	319.22	75.81	93.22	631.21	596.86
	(0.38)	(767.74)	(1,207.98)	(1,649.85)	(55.10)	(510.57)	(1,611.23)	(1,229.83)	(286.97)	(354.86)	(128.52)	(70.81)	(340.82)	(643.28)
DG + both	691.62	594.46	847.22	938.63	135.25	−191.80	561.28	86.85	−2.22	−174.67	54.49	39.70	397.24	168.27
	(0.17)	(597.53)	(918.74)	(1,397.71)	(119.23)	(301.57)	(1,523.50)	(623.14)	(84.80)	(178.22)	(113.26)	(58.99)	(311.46)	(471.40)
Observations	2,127	2,128	2,077	2,085	2,148	2,156	2,155	2,155	2,143	2,156	2,137	2,142	2,129	2,125
Control Group Mean, Year One	2,498	3,215	3,629	8,039	112	1,221	7,460	2,506	321	243		224		1,636
Control Group Mean, Baseline	1,248	4,566	7,552	4,243	196	479	803	638	256	258		156		874
Control Group Trend pvalue	(0.13)	(0.02)	(0.02)	(0.01)	(0.21)	(0.10)	(0.36)	(0.00)	(0.43)	(0.86)		(0.36)		(0.03)

*Panel C: Year Two, Any DG*	(1)${}^{‡}$	(2)	(3)	(4)	(5)${}^{‡}$	(6)${}^{‡}$	(7)${}^{‡}$	(8)${}^{‡}$	(9)${}^{‡}$	(10)${}^{‡}$	(11)	(12)	(13)	(14)

AnyDG		20.80							−163.76	20.75	26.42	−2.30	53.91	232.68
		(145.66)							(329.51)	(26.15)	(20.48)	(17.68)	(62.39)	(125.40)

*Panel D: Year Two, DG by Sub-Treatment*														

DG no messaging		−170.71							15.71	41.88	31.81	33.24	14.03	203.36
		(159.34)							(367.46)	(41.85)	(32.27)	(33.19)	(91.88)	(264.97)
DG + labor		678.27							−166.36	−41.49	8.52	−56.53	28.63	47.73
		(238.82)							(204.27)	(45.12)	(43.12)	(33.69)	(109.24)	(310.86)
DG + self-efficacy		87.86							−256.25	6.58	−10.29	−39.23	120.04	27.20
		(235.34)							(210.33)	(53.01)	(43.98)	(35.00)	(131.04)	(309.54)
DG + both		57.25							−253.66	−43.63	−16.40	−42.68	18.78	53.74
		(179.70)							(248.82)	(43.66)	(40.92)	(35.29)	(101.03)	(323.30)
Observations		1,913							2,039	2,047	2,054	2,056	2,053	2,053
R-squared		0.01							0.00	0.00	0.00	0.00	0.00	0.00
Control Group Mean, Year Two		1965							1618	221.5	106.4	135.4	415.4	773.1

Standard errors, clustered at the village level, p-values reported in parentheses. The effects of DG + labor, DG + self-efficacy and DG + both are the marginal effects relative to DG no messaging. **Inputs** is the amount spent in Indian Rupees on each input per acre in a season, and **Hired Labor Expenses** is the amount spent in Indian Rupees on hiring labor in a season on a specific practice. **SRI Relevant** refers to practices where we would expect differences in inputs between traditional and SRI grown rice. ${}^{‡}$ In Year Two we did not conduct a midline survey, thus, we did not survey farmers on their midline practices. Control Group Trend is Year One’s control group mean outcome minus the Baseline’s control group mean outcome, and the pvalue in parentheses reflects an unpaired ttest between the Baseline and Year One control group means. We control for whether a household’s total self-reported farm output aligns with the aggregate of their reported sold, consumed, stored, and shared paddy within a margin of error of 100 kilograms throughout.

**Table 8:** Mean Bayesian posterior distribution of treatment effects (per acre).

		Effect	HDIlo	HDIhigh	Prob <0	Prob >0
*Adoption Index*						

	DG no messaging	−0.01	−0.13	0.12	0.58	0.42
	DG + labor	0.003	−0.11	0.12	0.47	0.52
	DG + self_efficacy	0.06	−0.06	0.19	0.16	0.84
	DG + both	0.009	−0.11	0.12	0.43	0.57

*Labor Days*						

	DG no messaging	2.84	−4.99	10.9	0.24	0.76
	DG + labor	−2.48	−9.73	4.94	0.75	0.26
	DG + self_efficacy	−1.4	−8.97	6.36	0.64	0.36
	DG + both	−0.08	−7.47	7.22	0.51	0.49

*Expenditures* (Rps)						

	DG no messaging	−64.5	−913	806	0.56	0.44
	DG + labor	86.8	−769	930	0.42	0.58
	DG + self_efficacy	−48.5	−881	783	0.55	0.46
	DG + both	−137	−989	787	0.62	0.38

*Yield* (kgs/Acre)						

	DG no messaging	228	96.1	361	0	1.0
	DG + labor	75.6	−44.9	196	0.11	0.89
	DG + self_efficacy	0.725	−128	133	0.50	0.50
	DG + both	156	35.8	279	0.006	0.99

*Profits* (Rps)						

	DG no messaging	4470	1360	7700	0.002	0.99
	DG + labor	752	−1990	3520	0.30	0.70
	DG + self_efficacy	173	−2740	3020	0.46	0.54
	DG + both	3610	929	6340	0.004	0.99

The 95% Highest Density Interval (HDI) indicates the most likely estimated parameter values that comprise 95% of the distribution of possible effects with HDIlo and HDIhigh as the bounds. The Prob < 0 or > 0 is the probability that the true effect is less than or greater than zero.

**Fig. 6:**
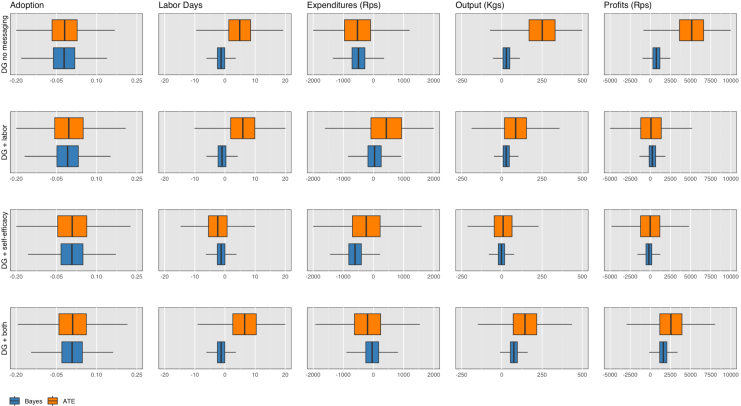
Posterior distribution of average treatment effect versus treatment effects from OLS per farm. Notes: Each boxplot represents the treatment effect for each treatment arm on the outcomes using a Bayesian model with weak priors versus OLS. For the Bayesian model, the thin line covers the central 95 percent posterior interval, the box covers the central 50 percent posterior interval, and the vertical bar within the box marks the posterior mean. For the OLS, the thin line covers the standard 95 percent confidence interval, the box covers a 50 percent confidence interval computed in the same way, and the vertical bar within the box marks the mean of the posterior.

Overall, the Bayesian estimation paints a clear picture that DG provides additive benefits to traditional NLRM training alone resulting in a 5%–12% increase in output, 12%–18% increase in yield and 9%–24% increase in estimated profits. Fig. 6, Fig. 10 graph the treatment effects from estimating the Bayesian model compared to average treatment effects. Note again, here the estimated treatment effects are for mutually exclusive treatment arms. The Bayesian estimation displays smaller and more precisely measured effects than the average treatment effects. In terms of inputs, the results differ by output per farm versus output per acre. The results indicate that per farm labor days and expenditures likely decreased for DG no messaging and DG + both, however, per acre, it does not appear that inputs declined, but yield, nevertheless, did. Our interpretation here is that if large farmers are gaining more in terms of output per farm they may also have increased their inputs more than small farmers. Once we control for the size of the farm this increase in inputs disappears.

**Fig. 7:**
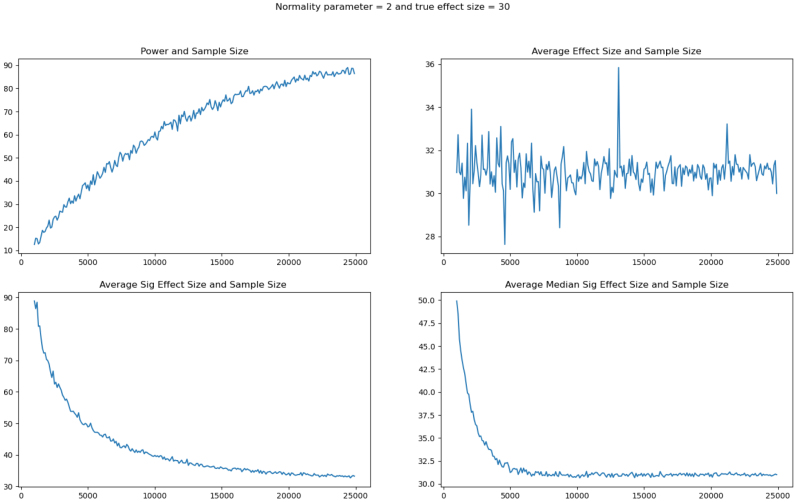
A simulation of power and statistically significant effect sizes on output. Notes: The above plots 1000 simulations for each sample size ranging from 1000 to 25,000. We use the modes of the posterior distribution of the parameters estimated in our Bayesian hierarchical model, namely a normality parameter of 2.0, a mean effect size of 30 where the mean and se of the control are 231 and 271, and the mean and se of the treated group are 244 and 316. As we increase N for each simulation of the control and treated data we store the estimated effect size, and statistically significant effects sizes from an OLS and median regressions. The graphs on the left show that 80 percent power is still not achieved with 25,000 observations, and that the true effect size of 30 is only recovered after 20,000 observations when the normality parameter is this low.

**Fig. 8:**
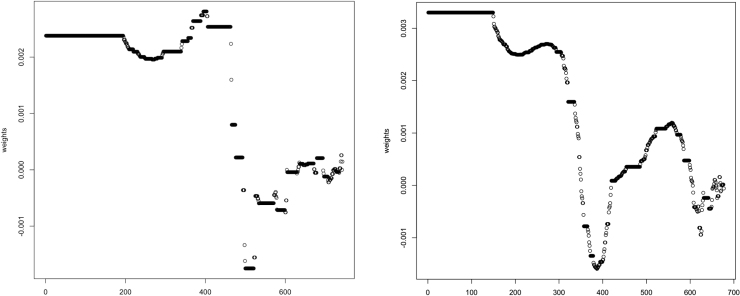
Weights from the weighted average quantile estimation of AnyDG. Notes: Each plot represents the estimated weights from the weighted average quantile treatment effect as described in Athey et al. (2023) for AnyDG, meaning the average effect for any of the DG treatment arms.

**Fig. 9:**
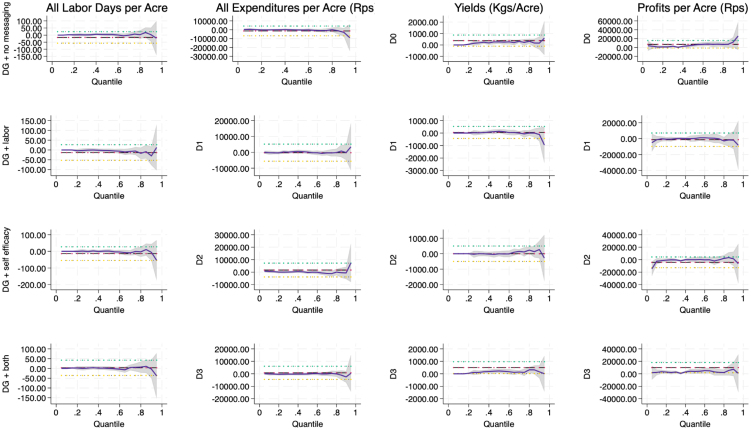
Quantile treatment effects of DG (per acre). Notes: Each plot represents the treatment effect (blue line) for each treatment arm on the outcomes using a quantile regression with the treatment effect size on the y-axis and each quantile (estimated at quantile intervals of 0.05) along the x-axis. The gray area represents 95% confidence, and the red, blue and yellow lines represent least squares estimate and its confidence interval. (For interpretation of the references to color in this figure legend, the reader is referred to the web version of this article.)

**Fig. 10:**
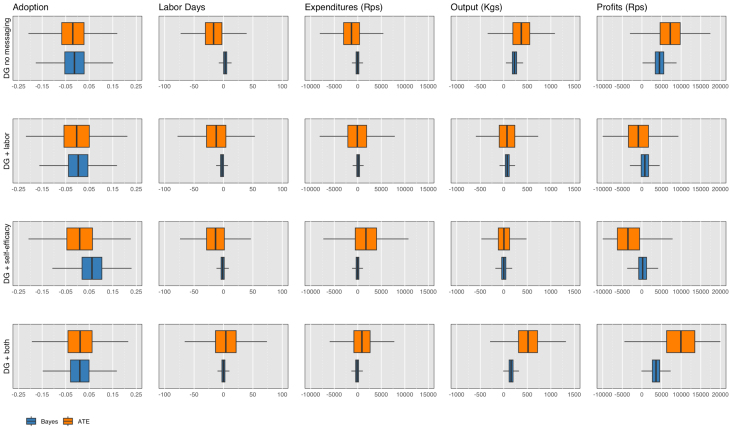
Posterior distribution of average treatment effect versus treatment effects from OLS (per acre). Notes: Each boxplot represents the treatment effect for each treatment arm on the outcomes using a Bayesian model with weak priors versus OLS. For the Bayesian model, the thin line covers the central 95 percent posterior interval, the box covers the central 50 percent posterior interval, and the vertical bar within the box marks the posterior mean. For the OLS, the thin line covers the standard 95 percent confidence interval, the box covers a 50 percent confidence interval computed in the same way, and the vertical bar within the box marks the mean of the posterior.

## 7 Discussion

We focus on three main points: the economic and statistical significance of the intervention on the main outcomes, output and yield, given each methodology; the mechanism of the change on output and yields given the lack of effect on adoption of the SRI practice; and the effect of the main DG arm without messaging compared to DG with labor and/or self-efficacy messages.

First, the above results from our intervention comparing average treatment effects, quantile regressions, weighted quantile regression and a Bayesian hierarchical model point to positive impacts on output per farm and per acre (yields). Both the main treatment arm of DG without messaging (for yields) and DG with both the labor and self-efficacy messages (for yields and output) have economically and statistically significant impacts. The percentage increase over mean starting values are in the range of 6%–30%, and this largely depends on the outcome (output versus yields) and method used: quantile regression, Bayesian model or weighted quantile regression. We look at both output and yields. We are interested in how the average farm fairs with the DG intervention (output), but we also want to control for the size of each farm, which can help with the precision of estimate treatment effects. If we look at output alone, the DG + both arm has both the largest economic and statistically significant effects. The median regression shows a 16% increase, and the mean of the Bayesian posterior, a 12% increase. The WAQ estimates a 6% increase, which is not statistically significant for output in levels, but is when output is transformed by the inverse hyperbolic sine function, which suggests that the non rice growers may be driving that difference. If we focus in on yields and the DG no messaging and DG + both arm, median regressions show a 18%–22% increase, the WAQ estimator shows a 6%–8% increase, and the mean of the Bayesian posteriors 12%–18% increase, all statistically significant or non-zero. Thus, what the three methodologies show is that the imprecisely measured ATE is likely due to imprecisely measured treatment effects in the upper tails.

We saw no movement on the adoption index but observed meaningful economic impacts on output and yield, albeit imprecisely measured. We argue that this is because the training videos encourage the same adoption of practices that the control was exposed to – which are relatively fixed for SRI. Rather, the purpose of DG aim is to improve the execution of each task. To the first point, Barrett et al. (2021) defines adoption as having practiced at least three of the six key SRI practices on at least one plot of land, which farmers did not report doing. Barrett et al. (2021) lists those practices as “transplanting younger (twenty-day-old) seedlings; (b) transplanting one to two seedlings per hill; (c) wide spacing of transplanted seedlings (25 × 20 cm); (d) providing organic matter amendments (e.g., compost, manure) to the soil; (e) following the AWD method of irrigation; and (f) mechanically weeding at regular intervals”. Both treatment and control groups report having adopted about one practice among the six SRI practices on average. Regarding the second point, since implementation of the practice is a key objective of DG, we might expect a change in the variance of outcomes in addition to the means, which we can quantify with the posterior estimates from the Bayesian model. We find that the mode of the variance of the posterior estimates increases for output and yields in addition to the means. For example, the difference in the modes of the posterior standard deviation estimates of treatment versus control for the DG no messaging arm on output (yield) is 42.6 (81.9), and 100% (94%) of the 95% HDI is above zero, meaning that the standard deviation or variance of the output outcome increased. For the DG + both outcome the latter figure is 74.6 (56.5) and 100% (77%) of the 95% HDI is above zero. For the DG + labor and DG + self-efficacy arms these effects are less pronounced.

Regarding the effects on intermediate inputs, both treatment and control groups received the same quantitative information regarding input use, and hence, we see almost no movement on inputs in the quantile regressions. The only instances where we a see a small drop in labor and expenditures is for the DG + self-efficacy arm per farm, which is consistent with our findings from the Bayesian model (results available upon request). We attribute this to the videos and repetition helping farmers better target their labor needs compared to standard extension training alone.

While we might not expect to see differences in input use between farmers who receive standard extension (the control) and farmers who receive standard extension with DG (treated groups) since both groups received the same information on SRI, we should expect to see a difference in input use between the year previous to the intervention. We check whether the control group arm’s use of inputs at midline is the same as their responses regarding inputs in the previous year. Table 7 breaks down the costs of SRI by input and practices. The Control Group Trend row summarizes information gathered at baseline about the previous year. The Control Group Mean, Year One summarizes information gathered in the first year of the intervention after the intervention was implemented. We know that SRI traditionally requires less irrigation and fertilizer as inputs, and requires more labor for land preparation, transplantation and weeding. Indeed, we see that the average differences in the control group from Baseline to Year One are statistically significant in all of the latter inputs and practices. In addition, the number of transplantation days increase from 9.8 days to 24.5 days per farm or 14.7 days, statistically significant at the 1% level. This is also in line with the one extra day required for transplantation per katha (1 katha = 0.04 acres) (which is relayed in our labor message). Namely, the average acreage towards rice is 0.8 acres and the median is 0.4, so an extra day per katha would translate to 10–20 more days of labor for the average farmers, which is about the increase we see between the previous year (retrospectively collected) and the year after which SRI was introduced to both the control and treated groups. Of course, these comparisons are not causally determined, since our entire sample was trained in SRI, but it does provide some indication that inputs and labor changed in a way that is consistent with farms having adopted SRI across all treatment arms, including the control.

Finally, we asked questions that tested participants’ knowledge of SRI-specific adoption practices, but *only* among the farmers who self-reported that they fully adopted SRI, which was only 13% of our treated sample. Among the treated, we saw a weakly statistically significant increase of 4% of self-reported adopters by Year One for the main treatment arm, but we know that these adopters can only partially, but not fully, explain the gains in output. Many other farmers who do not report full adoption of SRI saw gains as well. This is because, as noted earlier, SRI is not a fixed technology – farmers can adopt parts of the practices on parts of their field (Glover, 2011). Other studies also show that when farmers are exposed to SRI they only adopt 1–2 practices out of the 6 SRI practices (Barrett et al., 2021). Thus, we suspect that many farmers – control and treated – who are reporting having grown traditional rice, did adapt aspects of SRI practices (as evidenced by the changes in inputs and labor expenditures for the Control Group Trend).

Regarding the observed differences in the effects of the treatment arms, the primary improvements in output and yield are attributable to the DG no messaging arm and the DG + both messages arm. Conversely, the DG + self-efficacy arm and DG + labor arm exhibited no statistically significant effects. These findings imply that the impacts of the additional messages are not simply additive. Rather, they are effective through their complementarity with one another. A possible explanation for this result stems from the literature on self-efficacy. Despite self-efficacy being positively associated with goal choice, effort and persistence, Bandura (1985) notes that self-efficacy can be negatively correlated with planning and that some amount of self-doubt is, in fact, good in preparatory training contexts. This was later experimentally confirmed by Vancouver and Kendall (2006). Vancouver and Kendall (2006) shows that when individuals have high self-efficacy they report using less study time, and subsequently exhibit lower performance on exams. Thus, while self-efficacy may be important for setting goals, higher self-efficacy can reduce preparedness and skill acquisition for goal attainment, particularly in the training and learning setting. Thus, in our setting self-efficacy could have backfired without adequate preparedness. However, when the messages are combined, the two work in tandem to both prepare farmers of changes in their labor inputs as well as to encourage them that they will be able to complete the work.

In summary, what DG delivers is repeatable and standardized information, in addition to a means for adding on more nuanced messages in a quality controlled manner. Thus, while we should not expect inputs and adoption numbers to change between control and treated groups we should expect improvements in output per farm and output per acre if DG improves farmers’ implementation of SRI. In our specifications that address our fat tailed data, we do indeed see a statistically significant change in our output variables for the 25% quantile and median quantile. And the means of the posterior estimates from the Bayesian hierarchical model also demonstrate positive impacts along the same magnitudes.

## 8 Disperse outcomes and power

Disperse survey outcomes such as profits, output, yield, and expenditure among poor households are not uncommon in economics. Such dispersion could be because the data generating process is fat tailed with genuine extreme values or because of measurement error (Gollin and Udry, 2021). For example, Okorie et al. (2023) shows that the majority of crop yields across six different East African countries exhibit heavy tails and finds that a power law distribution best captures the upper tails. In the case of measurement error, potential solutions are to increase the frequency of collecting data rather than one retrospective question that covers decisions made over the course of several months (McKenzie, 2012). Gollin and Udry (2021) formally studies the contributions of input heterogeneity (including land quality), firm level shocks, measurement error and misallocation of inputs as sources of dispersion in yield in Uganda and Tanzania. They conclude that misallocation has been attributed too much importance in explaining dispersion in household level farm productivity: “Late-season production shocks and measurement error in factors of production and output together can account for about half to two-thirds of the variance in log productivity residuals” (Gollin and Udry, 2021).

**Fig. 11:**
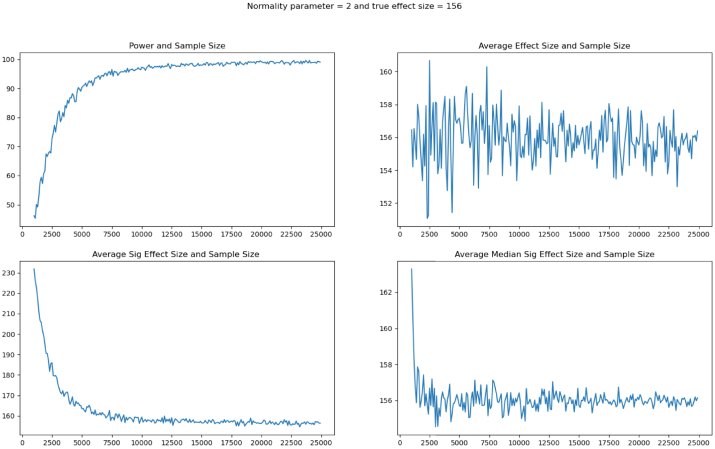
A simulation of power and statistically significant effect sizes on yield. Notes: The above plots 1000 simulations for each sample size ranging from 1000 to 25000. We use the modes of the posterior distribution of the parameters estimated in our Bayesian hierarchical model, namely a normality parameter of 2.0, a mean effect size of 156 where the mean and se of the control are 663 and 647, and the mean and se of the treated group are 819 and 702. As we increase N for each simulation of the control and treated data we store the estimated effect size, and statistically significant effects sizes from an OLS and median regressions. The graphs on the left show that 80 percent power is still not achieved with 3000 observations, and that the true effect size of 156 is only recovered after 8000 observations when the normality parameter is this low.

Given the disperse outcomes and disperse treatment effects in the right tails of the data, it is natural to consider that imprecisely measured average treatment effects could be remedied by having had a larger sample size ex-ante. Here we consider that notion via simulation. We consider what sample size we would have needed to achieve 80% power using the mean of the posterior distributions from the Bayesian analysis to detect a known effect size in output (yields), 30 kilograms per farm (156 kgs/acre); with a normality parameter of 2.0 (2.0); where the mode of the standard deviation in the control is 272 (647) and in the treatment 316 (702). In Fig. 7, Fig. 11, each data point is the average of 1000 simulations given a sample size ranging from 1000 to 25000. As we increase N for each simulation of the control and treated data we store the estimated effect size, and statistically significant effect sizes.

In Fig. 7 upper left, 80 percent power is only achieved with 25,000 observations for output, and in Fig. 11, 3000 for yield. The bottom left graphs are a depiction of the fact that under-powered studies typically will exhibit overstated effect sizes (Gelman and Carlin, 2014). Fig. 7, Fig. 11 show that with a known true effect size for output (yield) of 30 kgs (156 kgs/acre), sample should increase to 25,000 (8000) observations, a size that was not feasible in our (and many other) contexts. Thus, the simulation is an important reminder that increasing sample sizes to a point where we can recover the true effect size is potentially infeasible and potentially not the most cost-efficient approach to studying program impacts where the outcomes are fat tailed.

## 9 Cost-effectiveness

The marginal cost of the DG arm is 96 Rps per farmer.^17^ The marginal benefits vary widely as discussed above, but are consistently well above the marginal cost. For the DG with no messaging subtreatment arms, marginal benefits are 5224 Rps, 1282 Rps, and 590 Rps from the average, median, and Bayesian approaches, respectively. The same for the DG+both messaging subtreatments are 2386 Rps, 2026 Rps, and 1620 Rps. The equivalent for the arms with exactly one of the messaging arms, as discussed above, are not statistically significantly different than zero.^18^ Thus the DG arm (as long as it includes the messaging, or no messaging) is cost-effective relative to the control (i.e., human-only extension), in that the improvement in profits, irrespective of which estimation is used, exceeds the marginal cost.

**Table A.1:** Baseline summary statistics per household, by treatment assignment means and standard errors.

VARIABLE	Control	DG ⋅ only	DG ⋅ labor	DG ⋅ self-efficacy	DG ⋅ both	F-stat p-value
	(1)	(2)	(3)	(4)	(5)	(6)
Heard of SRI (y/n)	0.33	0.30	0.28	0.33	0.32	0.97
	(0.03)	(0.04)	(0.05)	(0.05)	(0.04)	
Adoption Index	1.40	1.45	1.51	1.53	1.32	0.74
	(0.07)	(0.08)	(0.10)	(0.09)	(0.08)	
Paddy Acres	1.71	1.84	2.08	1.75	1.82	0.91
	(0.12)	(0.15)	(0.29)	(0.16)	(0.12)	
Expenditures 000’s Rps	10.16	11.20	14.45	9.38	15.22	0.57
	(1.09)	(1.75)	(3.16)	(2.69)	(3.89)	
Rice Output 00’s KGs	6.04	7.00	5.43	7.31	6.14	0.81
	(0.63)	(0.82)	(0.80)	(1.22)	(0.57)	
Estimated Profit 000’s Rps	1.32	1.16	-5.35	5.40	-3.34	0.26
	(1.56)	(2.14)	(3.54)	(3.56)	(3.93)	
	232	135	94	108	149	

Standard errors, clustered at the village level, reported in parentheses. All agricultural outcomes for the baseline are retrospective reports for the previous year’s kharif season in 2013. The baseline was compromised due to mud slides and difficulty reaching more than half of the targeted sample. **Expenditures** represents the common set of total expenditures between Baseline Year One and Year Two including fertilizer, machine rentals, and irrigation for the entire season. For the full set of expenditures and labor days surveyed by year see Table 7. **Rice Output** is the total amount of rice produced in kilograms for 2013. **Estimated Profit** is a constructed variable and is equal to harvest storedx$p_{r}$ + harvest soldx$p_{f}$ – total expenditures – hired labor, where $p_{f}$ is the reported farm gate price at which a farmer sold her rice, and $p_{r}$ is the estimated retail price (24.2 Rps/kgs from Bhoi et al. (2019)). Column 6 reports the p-value from a F-test of the equality of the coefficients in each row.

**Table A.2:** Orthogonality Check: Household demographics and dwelling characteristics from midline survey year one, means and standard errors.

VARIABLE	Control	DG no messaging	DG ⋅ labor	DG ⋅ self-efficacy	DG ⋅ both	F-stat p-value
	(1)	(2)	(3)	(4)	(5)	(6)
*Baseline*						

Hhd size	7.33	6.66	7.53	7.7	7.54	0.06
	(0.22)	(0.22)	(0.35)	(0.31)	(0.29)	
SHG Membership	3.35	3.16	3.66	3.72	3.3	0.47
(years)	(0.13)	(0.17)	(0.22)	(0.14)	(0.13)	
Age of home	13.62	15.15	12.86	14.56	14.53	0.79
	(0.90)	(1.26)	(1.36)	(1.38)	(1.29)	
Rooms in house	3.12	2.99	2.61	2.96	3.23	0.06
	(0.13)	(0.14)	(0.13)	(0.14)	(0.16)	
Has electricity (y/n)	0.48	0.49	0.43	0.46	0.45	0.98
	(0.03)	(0.04)	(0.05)	(0.05)	(0.04)	
Grass thatched roof (y/n)	0.34	0.45	0.44	0.37	0.38	0.62
	(0.03)	(0.04)	(0.05)	(0.05)	(0.04)	
Mud walls (y/n)	0.35	0.36	0.5	0.33	0.34	0.28
	(0.03)	(0.04)	(0.05)	(0.05)	(0.04)	
Owns irrigation pump (y/n)	0.08	0.13	0.04	0.08	0.1	0.19
	(0.02)	(0.03)	(0.02)	(0.03)	(0.02)	
Owns tractor (y/n)	0.04	0.03	0.01	0	0.02	0.00
	(0.01)	(0.01)	(0.01)	(0.00)	(0.01)	
Observations	232	135	94	108	149	

*Midline*						

Hhd size	7.08	6.69	6.84	7.17	6.94	0.19
	(0.12)	(0.16)	(0.15)	(0.19)	(0.17)	
SHG Membership	3.52	3.51	3.64	3.59	3.46	0.90
(years)	(0.06)	(0.09)	(0.08)	(0.09)	(0.09)	
Age of home	13.6	16.06	15.43	13.97	15.57	0.29
	(0.54)	(0.95)	(0.95)	(0.88)	(0.90)	
Rooms in house	3.19	3.27	2.95	3.11	3.33	0.11
	(0.07)	(0.11)	(0.09)	(0.09)	(0.11)	
Has electricity (y/n)	0.45	0.49	0.47	0.48	0.49	0.93
	(0.02)	(0.03)	(0.03)	(0.03)	(0.03)	
Grass thatched roof (y/n)	0.3	0.37	0.32	0.32	0.3	0.35
	(0.02)	(0.03)	(0.02)	(0.03)	(0.02)	
Mud walls (y/n)	0.3	0.34	0.31	0.32	0.31	0.94
	(0.02)	(0.03)	(0.02)	(0.03)	(0.02)	
Owns irrigation pump (y/n)	0.12	0.12	0.12	0.12	0.14	0.93
	(0.01)	(0.02)	(0.02)	(0.02)	(0.02)	
Owns tractor (y/n)	0.01	0.01	0.02	0.01	0.02	0.34
	(0.00)	(0.00)	(0.01)	(0.01)	(0.01)	
Observations	743	340	354	340	379	
						

*** p<0.01, ** p<0.05, * p<0.1 Standard errors, clustered at the village level, reported in parentheses. The Baseline was compromised due to mud slides and difficulty reaching more than half of the targeted sample. Since Year One’s midline sample was substantially larger than the baseline, this table checks for balance between treatment and control groups on durable assets and household size. Column 6 reports the p-value from a F-test of the equality of the coefficients in each row.

## 10 Conclusion

We present experimental evidence on the effects of a video-based intervention that trains female farmers in the presence of an already existing traditional extension training infrastructure, the National Rural Livelihood Mission (NRLM). We worked with a large non-profit organization, Digital Green, in Bihar, India, which has reached over 5.6 million farmers through their video aids.

As is common with agricultural data, our outcomes exhibit fat tails (Okorie et al., 2023), which make average treatment effect estimates unreliable. This is reflected in the large and imprecisely measured average treatment effects of our intervention. Quantile regressions show that this imprecision is driven by the upper tails of the outcomes. A more recent technique, weighted quantile regressions (Athey et al., 2023), shows that the observations above the median in our data are particularly noisy and puts little weight on the effects in the upper tails to estimate a more precise average treatment effect.

One might think that a larger sample size would remedy the problem of our imprecisely measured average treatment effects. However, if the normality parameter, which controls the heaviness of the tails in a t-distribution, of the data generating process is too small then the variance of the data is essentially infinite. Any estimation of that variance using sample data will be biased, and extreme values will still be common enough that they can meaningfully affect the mean. We show through simulation that the increase in the sample size necessary to power a study like ours would be infeasible — both because it could exceed the actual population size and/or because it would be prohibitively costly to collect. In summary, we show that when data exhibit fat tails average treatment effects can obfuscate meaningful impacts. Alternative methodologies should be considered that can accommodate the underlying data patterns, address data dispersion, or estimate treatment effects across the entire data distribution.

We use two different methods to address fat tails, quantile regressions and a Bayesian hierarchical model. Quantile regressions allow us to estimate how different points of the outcome distribution change with the intervention, whereas a Bayesian hierarchical model allows us to study the entire probability distribution of the treatment effect while accounting for fat tails via priors. The results from Bayesian estimation with varied priors points to non-zero effects, where the mean of the posterior distributions are of the same order of magnitude as the point estimates between the 25% and 50% quantile regressions. Overall, the effect sizes range from a 6%–16% increase over baseline outcomes for output, and a 25%–31% increase over baseline outcomes for yields. We find that the added messages regarding labor requirements and self-efficacy, issues highlighted by the literature for adoption of SRI and of new technologies more generally, had a complementary effect. The DG arms with just one of the messages – labor or self-efficacy – did not have statistically significant impacts, and adding the self-efficacy message alone actually reduced the effectiveness of DG. We argue that the self-efficacy message could potentially lead farmers to be confident yet under prepared without the added information of the labor message. An area for future research is to unpack the mechanism of the self-efficacy message and understand if there is self-efficacy content that would not detract from the effects of the main treatment arm, and with what type of information messages it can be effectively paired. This should also be studied in the context of self help groups, and whether self help groups help to activate the effects of self-efficacy messaging where fellow farmers or VRPs may serve as reference points.

We find little to no effects of DG on inputs – labor and expenditures – both in quantile regressions and the Bayesian model. We attribute this to both treatment and control being trained in SRI via standard extension training. Only the treatment group received repeated information via video. There is also no movement in overall adoption from the DG interventions compared to the control group. There are several reasons for why adoption can be low and yet there are impacts on output and yields. First, in addition to the control group having also received SRI training, we also did not capture adoption adequately. First, we only asked respondents who fully adopted SRI to reply to questions about SRI practices, and yet, partial adoption was likely and is traditionally common for SRI farmers. In particular, our questions related to SRI practices were answered by very few farmers (only 13% among the treated), and were predicated upon a farmer saying that she adopted SRI fully. However, we suspect that many farmers adopted only aspects of SRI and only on portions of their plots. Future survey questions on SRI adoption should be constructed in such a way that they can be posed to all farmers. This would also help us determine which aspects of SRI are adopted initially, and what practices are more likely to be disadopted over time. Second, we did not adequately monitor the degree to which farmers correctly implemented practices, which is, ultimately, what it means to adopt SRI. Our adoption index is constructed from a series of questions about the practices and correct responses associated with SRI. Farmers in both groups may report adopting the practices, but DG could help them improve in their execution of practice, and this is something our index cannot capture. Such a verification would require either real time on the ground verification or verification through imagery of the plots, which is an area for future research.

## CRediT authorship contribution statement

**Tushi Baul:** Data curation. **Dean Karlan:** Conceptualization, Methodology, Writing – review & editing. **Kentaro Toyama:** Conceptualization, Methodology, Writing – original draft, Writing – review & editing. **Kathryn Vasilaky:** Conceptualization, Data curation, Formal analysis, Methodology, Writing – original draft, Writing – review & editing.

## Figures and Tables

**Table A.3:** ITT treatment effects on farm adoption practices for traditional growers.

VARIABLES	No.	Age	No.	Seedling	Row
	Weedings	Sapling	Saplings	Spacing	Spacing
*Panel A: Year One, Any DG*	(1)	(2)	(3)	(4)	(5)

Any DG	−0.02	−0.05	0.27	0.02	0.00
	(0.68)	(0.93)	(0.27)	(0.66)	(0.75)

*Panel B: Year One, DG by Sub-Treatment*	(5)	(6)	(7)	(8)	(9)

DG no messaging	−0.03	−0.88	0.02	0.00	−0.00
	(0.58)	(0.32)	(0.90)	(0.97)	(0.73)
DG + labor	0.04	0.08	0.79	−0.01	0.01
	(0.62)	(0.95)	(0.34)	(0.84)	(0.52)
DG + self-efficacy	0.06	1.34	0.08	0.02	0.00
	(0.43)	(0.16)	(0.75)	(0.75)	(0.80)
DG + both	−0.03	1.83	0.10	0.05	0.01
	(0.63)	(0.03)	(0.61)	(0.40)	(0.44)
Observations	1,726	1,697	1,659	1,721	1,726
Control Group Mean, Year One	1.66	24.13	2.82	0.44	0.03
Control Group Trend	−0.24	−0.09	−0.15	0.33	−0.35

Standard errors, clustered at the village level, p-values reported in parentheses. The effects of DG + labor, DG + self-efficacy and DG + both are the marginal effects relative to DG no messaging. **No. Weedings** is “How many times did you weed your field?” At least two times weeding are required for a SRI field in Kharif season. **Age Sapling** is “What was the age of the sapling when you transplant to nursery bed?” Saplings should be 8–14 days old for transplantation. **No. Saplings** is “How many saplings did you transplant together?” One sapling should be planted at a dip. **Seedling Spacing** is “Was there a fixed distance between each seedling? ” 8–10 inches distance between saplings. **Row Spacing** is “Was there a fixed distance between each seedling? ” 8–10 inches distance between rows. Control Group Trend is the Year One’s mean control group outcome minus the baseline’s mean control group outcome. Control Group Trend is Year One’s control group mean outcome minus the Baseline’s control group mean outcome. We control for whether a household’s total self-reported farm output aligns with the aggregate of their reported sold, consumed, stored, and shared paddy within a margin of error of 100 kilograms throughout.

**Table A.4:** Process Verification: Self-reports on Watching videos from Digital Green.

VARIABLES	Seed	Nursery	Trans
	Video	Video	Video
*Panel A: Year One, Any DG*	(1)	(2)	(3)

Any DG	0.14	0.13	0.17
	(0.00)	(0.00)	(0.00)

*Panel B: Year One, DG by Sub-Treatment*	(1)	(2)	(3)

DG no messaging	0.17	0.14	0.18
	(0.00)	(0.00)	(0.00)
DG + labor	−0.03	−0.02	−0.03
	(0.46)	(0.62)	(0.54)
DG + self-efficacy	−0.03	−0.02	0.00
	(0.46)	(0.58)	(0.96)
DG + both	−0.04	0.01	0.01
	(0.35)	(0.84)	(0.90)
Observations	1,739	1,739	1,739
Control Group Mean, Year One	0.05	0.03	0.08

Standard errors, clustered at the village level, p-values reported in parentheses. The effects of DG + labor, DG + self-efficacy and DG + both are the marginal effects relative to DG no messaging. **Seed Video**, **Nursery Video**, and **Trans Video** are binary response variables to whether the respondent said that he/she was exposed to any projected videos on Seed Treatment, Nursery Bed Preparation, or Transplantation in the past year. We control for whether a household’s total self-reported farm output aligns with the aggregate of their reported sold, consumed, stored, and shared paddy within a margin of error of 100 kilograms throughout.

**Table A.5:** Mean Bayesian posterior distribution of treatment effects (levels), strong priors.

		Effect	HDIlo	HDIhigh	Prob <0	Prob >0
*Adoption Index*						

	DG no messaging	−0.02	−0.13	0.09	0.64	0.36
	DG + labor	0.008	−0.13	0.12	0.56	0.44
	DG + self_efficacy	0.007	−0.11	0.13	0.45	0.55
	DG + both	0.08	−0.10	0.12	0.44	0.55

*Labor Days*						

	DG no messaging	−1.29	−4.79	2.23	0.76	0.23
	DG + labor	−0.96	−4.7	2.82	0.70	0.30
	DG + self_efficacy	−1.26	−4.95	2.34	0.75	0.25
	DG + both	−1.28	−4.79	2.29	0.76	0.24

*Expenditures* (Rps)						

	DG no messaging	−495	−1110	118	0.94	0.05
	DG + labor	37.7	−612	668	0.46	0.55
	DG + self_efficacy	−608	−1200	−3.12	0.97	0.02
	DG + both	−40.6	−668	576	0.55	0.45

*Output* (kgs)						

	DG no messaging	28.8	−31.3	90.7	0.17	0.82
	DG + labor	27.4	−28	82.6	0.17	0.83
	DG + self_efficacy	−2.57	−59.6	54.2	0.54	0.46
	DG + both	75.1	13.4	138	0.08	0.99

*Profits* (Rps)						

	DG no messaging	767	−491	2020	0.11	0.88
	DG + labor	261	−905	1470	0.34	0.66
	DG + self_efficacy	−173	−1210	886	0.63	0.37
	DG + both	1620	344	2900	0.05	0.99

The 95% Highest Density Interval (HDI) indicates the most likely estimated parameter values that comprise 95% of the distribution of possible effects with HDIlo and HDIhigh as the bounds. The Prob < 0 or > 0 is the probability that the true effect is less than or greater than zero.

**Table A.6:** Mean Bayesian posterior distribution of treatment effects (per acre), strong priors.

		Effect	HDIlo	HDIhigh	Prob <0	Prob >0
*Adoption Index*						

	DG no messaging	−0.01	−0.13	0.10	0.59	0.41
	DG + labor	0.003	−0.11	0.12	0.48	0.52
	DG + self_efficacy	0.06	−0.06	0.18	0.16	0.84
	DG + both	0.01	−0.10	0.12	0.43	0.57

*Labor Days*						

	DG no messaging	2.89	−5.15	10.7	0.24	0.76
	DG + labor	−2.49	−9.79	4.99	0.75	0.25
	DG + self_efficacy	−1.42	−8.97	6.34	0.64	0.36
	DG + both	0.06	−7.37	7.44	0.51	0.49

*Expenditures* (Rps)						

	DG no messaging	−60.6	−908	809	0.56	0.44
	DG + labor	86.8	−771	927	0.42	0.58
	DG + self_efficacy	−49.5	−906	777	0.55	0.45
	DG + both	−138	−1000	765	0.62	0.38

*Yield* (kgs/Acre)						

	DG no messaging	228	95.6	360	0	1.0
	DG + labor	75.2	−47.6	193	0.11	0.90
	DG + self_efficacy	0.99	−130	130	0.50	0.50
	DG + both	156	32.7	279	0.07	0.99

*Profits* (Rps)						

	DG no messaging	3600	940	6330	0.04	0.99
	DG + labor	3600	940	6330	0.04	0.70
	DG + self_efficacy	3600	940	6330	0.04	0.70
	DG + both	3600	940	6330	0.04	0.70

The 95% Highest Density Interval (HDI) indicates the most likely estimated parameter values that comprise 95% of the distribution of possible effects with HDIlo and HDIhigh as the bounds. The Prob < 0 or > 0 is the probability that the true effect is less than or greater than zero.

**Table B.1:** Videos.

*SRI Seed Treatment* Video discusses how to care for seeds including seed selection using a saline water solution; seed washing with fresh water 8--10 h; seed treatment with bebisteen powder for 18--20 h; and seed storage in moist sacks. A one acre plot requires 100 grams of seed as compared to the traditional method, which would require 1 kilogram of seed.

*Nursery Bed Preparation* Video discusses how to prepare raised nursery beds for seed planting seeds. The beds should be 10 ft in length, 3 ft wide, and 6 inches high, and situated next to the farmer’s paddy field. The beds should contain biomass mixed with the soil. The seeds are planted on the raised nursery beds with a spacing of 1-inch between rows.

*SRI Seeding and Transplantation* Video discusses the process of uprooting saplings and re-planting them in the field. Saplings are transplanted 8–10 days after nursery bed preparation. Fertilizer is applied prior to (6 kgs of biomass transplantation, and saplings are planted 10 cm apart. Watering and timing of watering are discussed.

The *Labor Message* describes the number of labor days required for the SRI method and the gains in output from SRI. The *Self-Efficacy Message* describes a farmer’s success with SRI, how he/she started applying SRI to part of a plot at first and then increased his/her coverage each year, and that he/she felt supported in his or her adoption of the technique by Village Representative Persons (VRPs).

**VIDEO ACTOR INFORMATION**

Keeping with DG’s efforts to include actors similar to video viewers, all video protagonists were female, aged 30–45, and belonging to the government’s category of Other Backward Class (OBC).

## Data Availability

Data will be posted to public repository.

## Appendix A

See Table A.1, Table A.2, Table A.3, Table A.4, Table A.5, Table A.6.

## Appendix B Videos and scripts

**Script for Clip (a)** Below is the script for Clip (a), a short (30–60) seconds) clip that shows the actual labor costs of SRI per unit area (e.g., katha).

Video producers should feel free to revise the language, tone, labor-cost information, and actor. The goal is a short, informative clip that indicates what the labor costs of SRI actually are on a per-area basis.

Recommended actor type: Same actor as in current videos– a woman who has successfully tried SRI in the past.

Dress: Actor is dressed in normal clothes worn for farming activities.

Background: Actor is standing in front of a neutral background (such as a house, or a farm, or a forest). They look directly into the camera when speaking.

Script: “Our household has been using SRI for paddy for several years. At first, we were worried that it would take more labor than traditional paddy, so we tried it in a corner of one of our plots of land. For [topic of the video], we found that SRI requires about [X more/less] days of labor compared with the traditional method. And over the entire planting cycle, there was only a total of [one day more] of labor per katha for SRI over traditional. For that we gained over [50 kg] more rice per katha with SRI, which even at [Rs. 10] per kg would be [Rs. 500] more in value per katha. Because of this, SRI is worth the little bit of extra labor, and we will plant using SRI again this year”.

Instructions to VRPs: After this section of the video plays, the VRPs should pause the video and ask the audience questions about the labor costs of SRI, both for this topic, and overall for SRI.

**Script for Clip (b)**

Below is the suggested script for Clip (b), a short (30–60 s) clip in which a local farmer who has implemented SRI mentions that she has successfully implemented SRI.

Video producers should feel free to revise the language, tone, and details of the content. The goal is a short, testimonial clip in which someone who was initially uncertain of his/her SRI-planting ability is now confident of his/her ability and encourages others to try it.

Recommended actor type: Same actor as in current videos, a woman who has successfully tried SRI in the past.

Dress: Actor is dressed in normal clothes worn for farming activities.

Background: Actor is standing in front of his/her well-stocked store of rice, or in front of a neutral background (such as a house, or a farm, or a forest). She looks directly into the camera when speaking.

Script: “Our household has been using SRI for paddy for several years. At first, I was not sure if we could plant SRI, because the technique is different from the traditional method. So, we tried it in a corner of one of our plots of land to begin with. We found that planting using SRI is not that different from the traditional method. We also found that the VRPs are very helpful in keeping us informed. We asked them questions from time to time and their answers helped us. Every year, we have had a larger harvest with SRI than we did with the traditional method. And now we know that we are able to plant using SRI. My family is a regular farming family– if our household can plant with SRI, then your household can also plant with SRI”.

Instructions to VRPs: After this section of the video plays, the VRPs should pause the video and do the following: First, ask the audience to repeat the last sentence of this section out loud and in unison, as follows: “If that woman’s family can plant with SRI, then my household can plant with SRI, too”. Second, ask if anyone in the audience has implemented SRI successfully. If there is such a person, that person should be invited to share their experience with the group. After that, again ask everyone to recite in unison, “If her family can plant with SRI, then my household can plant with SRI, too”.

## Footnotes

1 We do not have a treatment arm that received no training at all as NRLM extension training was already active in all the rice-growing districts of Bihar. For an evaluation of NRLM alone, which focuses on other household outcomes – e.g. income, consumption, credit access – see Kochar et al (2020) as well as Anon (2021) and Datta (2015) for its impacts on debt relief and restructuring household debt.

2 See DellaVigna and La Ferrara (2015) for a review, and Anon (2015) for an overview of edutainment projects in international development.

3 https://data.worldbank.org/.

4 For an in-depth overview of agricultural extension in India see Glendenning et al. (2010).

5 http://icrier.org/pdf/Agriculture-Extension-System-in-India-2018.pdf.

6 Ministry of Rural Development -- Letter on SHGs to the Government of India.

7 India Environmental 2015 Report, pg 14.

8 National Institution for Transforming India Water Index Report, pg 17.

9 Cornell University. Barrett et al. (2021) shows yield increases of 14%–17%, Thakur et al. (2011) finds a 32 percent increase, Kumar and Talati (2007) finds a 48 percent increase and Takahashi and Barrett (2013) finds a 64 percent increase.

10 RCT registered at Social Registry 313.

11 The data were collected by the Jameel Poverty Action Lab South Asia.

12 Examples of Digital Green’s videos can be found here.

13 One katha equals 0.4 acres.

14 The study was pre-registered at Social Science Registry, Trial AEARCTR-0000313.

15 A student t with normality parameter 30 is approximately normal.

16 The library used for the Bayesian analysis can be found at https://rdrr.io/cran/BEST/man/BEST-package.html.

17 67 Rps per farmer for the VRP (each VRP trains 10 farmers and costs 670 Rps) plus 29 Rps per farmer for the video screening (each video screening trains 3 farmers and costs 285 Rps); thus total marginal cost = 67+29 = 96 Rps per farmer. This 96 Rps per farmer is on top of the 285 Rp per farmer base cost of the training, i.e., the cost of the control group, which is irrelevant for the cost-effectiveness calculation for DG. The 285 Rps per farmer derived as follows: 1050 Rps for a VRP for 10 people, thus 105 for 1 VRP and 180 Rps per farmer in training costs; 105+180=285 per farmer.

18 Bayesian posteriors are displayed in Fig. 6.
